# Human chorionic villous mesenchymal stem/stromal cells protect endothelial cells from injury induced by high level of glucose

**DOI:** 10.1186/s13287-018-0984-0

**Published:** 2018-09-21

**Authors:** Y. S. Basmaeil, A. M. Al Subayyil, T. Khatlani, E. Bahattab, M. Al-Alwan, F. M. Abomaray, B. Kalionis, M. A. Alshabibi, A. S. AlAskar, M. H. Abumaree

**Affiliations:** 1 Stem Cells and Regenerative Medicine Department, King Abdullah International Medical Research Centre, King Abdulaziz Medical City, Ministry of National Guard Health Affairs, P.O. Box 22490, Mail Code 1515, Riyadh, 11426 Saudi Arabia; 2 National Center for Stem Cell Technology, Life Sciences and Environment Research Institute, King Abdulaziz City for Science and Technology, P.O. Box 6086, Riyadh, 11442 Saudi Arabia; 3 Stem Cell and Tissue Re-Engineering Program, King Faisal Specialist Hospital and Research Centre, Collage of Medicine, Al-Faisal University, MBC-03, P.O. Box 3354, Riyadh, 11211 Saudi Arabia; 4 Division of Obstetrics and Gynecology, Department of Clinical Science, Intervention and Technology, Karolinska Institutet, 14186 Stockholm, Sweden; 5 Center for Hematology and Regenerative Medicine, Karolinska Institutet, 14186 Stockholm, Sweden; 6 Department of Maternal–Fetal Medicine Pregnancy Research Centre and University of Melbourne Department of Obstetrics and Gynaecology, Royal Women’s Hospital, Parkville, VIC 3052 Australia; 7 College of Medicine, King Saud Bin Abdulaziz University for Health Sciences, King Abdulaziz Medical City, Ministry of National Guard Health Affairs, P.O. Box 3660, Mail Code 3124, Riyadh, 11481 Saudi Arabia; 8 Adult Hematology and Stem Cell Transplantation, King Abdulaziz Medical City, Ministry of National Guard Health Affairs, P.O. Box 22490, Mail Code 1515, Riyadh, 11426 Saudi Arabia; 9 College of Science and Health Professions, King Saud Bin Abdulaziz University for Health Sciences, King Abdulaziz Medical City, Ministry of National Guard Health Affairs, P.O. Box 3660, Mail Code 3124, Riyadh, 11481 Saudi Arabia

**Keywords:** Placenta, Chorionic villous mesenchymal stromal cells, Endothelial cells, Glucose, Proliferation, Migration, Monocyte invasion, Endothelium permeability, Gene expression

## Abstract

**Background:**

Mesenchymal stem/stromal cells derived from chorionic villi of human term placentae (pMSCs) protect human endothelial cells from injury induced by hydrogen peroxide (H_2_O_2_). In diabetes, elevated levels of glucose (hyperglycaemia) induce H_2_O_2_ production, which causes the endothelial dysfunction that underlies the enhanced immune responses and adverse complications associated with diabetes, which leads to thrombosis and atherosclerosis. In this study, we examined the ability of pMSCs to protect endothelial cell functions from the negative impact of high level of glucose.

**Methods:**

pMSCs isolated from the chorionic villi of human term placentae were cultured with endothelial cells isolated from human umbilical cord veins in the presence of glucose. Endothelial cell functions were then determined. The effect of pMSCs on gene expression in glucose-treated endothelial cells was also determined.

**Results:**

pMSCs reversed the effect of glucose on key endothelial cell functions including proliferation, migration, angiogenesis, and permeability. In addition, pMSCs altered the expression of many genes that mediate important endothelial cell functions including survival, apoptosis, adhesion, permeability, and angiogenesis.

**Conclusions:**

This is the first comprehensive study to provide evidence that pMSCs protect endothelial cells from glucose-induced damage. Therefore, pMSCs have potential therapeutic value as a stem cell-based therapy to repair glucose-induced vascular injury and prevent the adverse complications associated with diabetes and cardiovascular disease. However, further studies are necessary to reveal more detailed aspects of the mechanism of action of pMSCs on glucose-induced endothelial damage in vitro and in vivo.

## Background

Diabetes is a metabolic disorder characterized by hyperglycaemia, insulin resistance, and relative insulin deficiency [1]. Diabetes is associated with vascular complications that contribute to morbidity and mortality in diabetic patients [2–4]. In diabetic patients, hyperglycaemia stimulates the production of reactive oxygen species in the endothelium, which play an essential role in the development of vascular damage and contribute to the incidence of thrombotic events [5–8]. Indeed, diabetes shares similar features with cardiovascular diseases, which also features enhanced responses of inflammatory cells and increased formation of thrombosis because of endothelial cell dysfunction [9, 10].

Injured endothelial cells express elevated levels of adhesion molecules and have enhanced permeability [9, 10]. These two events stimulate the recruitment of immune cells, such as monocytes, as well as the entry of low-density lipoprotein cholesterol (LDL) from the blood vessel lumen into the wall [5–8]. Subsequently, LDL is oxidized to ox-LDL and is taken up by macrophages, which leads to the formation of foam cells, increases inflammatory responses, and leads to the deposition of collagen [5–8]. These events lead to the formation of atherosclerotic plaques, and the subsequent rupture of these plaques activates platelets and culminates in thrombosis [5–8]. Therefore, improving or alleviating the effects of endothelial cell damage in diabetes is a potential therapeutic target, with the expected outcome of repairing vascular dysfunction and preventing complications associated with diabetes, such as thrombosis and atherosclerosis.

Mesenchymal stem cells (MSCs) are multipotent stromal cells that are isolated from adult and fetal tissues, such as placenta [11]. Previously, we isolated MSCs from the chorionic villi of human term placentae (pMSCs) and reported their unique ability to regulate many of the critical cellular functions of their target cells [11]. Moreover, pMSCs show immunosuppressive properties that make allogeneic transplantation possible [12, 13]. Recently, we reported that pMSCs protect endothelial cells from damage induced by an oxidative stress mediator (i.e. hydrogen peroxide) [14]. Therefore, pMSCs have the key functional properties that make them a promising therapeutic tool for treating inflammatory diseases.

Here, we initially examined the ability of pMSCs to protect various important endothelial cell functions from oxidative stress induced by another oxidative stress mediator; glucose. To better understand the mechanism of endothelium damage repair following exposure to glucose and pMSC treatment, we investigated gene expression changes in a panel of endothelial genes that mediate important cellular functions. We report that pMSCs protect particular endothelial cell functions (i.e. proliferation, migration, permeability, and tube network formation (angiogenesis)) from glucose. In addition, pMSCs modify the effect of glucose on the expression of many genes that mediate endothelial cell functions. These data suggest that pMSCs have a protective function on endothelial cells in an oxidative stress environment induced by glucose. Thus, pMSCs are promising candidates for a stem cell-based therapy to repair endothelial cell injury induced by high glucose, and prevent complications associated with this injury. However, further studies are required to reveal more detailed aspects of the mechanism of action of pMSCs on glucose-induced endothelial damage both in vitro and in vivo.

## Methods

### Ethics of experimentation and collection of human placentae and umbilical cords

The study was approved by the institutional research board (Reference # IRBC/246/13) at King Abdulla International Medical Research Centre (KAIMRC), Saudi Arabia. Samples (i.e. placentae and umbilical cords) were obtained from uncomplicated human pregnancies (38–40 gestational weeks) following informed patient consent, and then processed immediately. All clinical and experimental procedures were performed in compliance with KAIMRC research guidelines and regulations.

### Isolation and culture of pMSCs

MSCs from the chorionic villi of human term placenta (pMSCs) were isolated using our published method [11]. Briefly, small pieces (~ 40 mg total wet weight) of the chorionic villi were washed thoroughly with sterile phosphate buffered saline (PBS, pH 7.4) and then incubated in a solution of DMEM-F12 (Life Technologies, Grand Island, NY, USA) containing 2.5% trypsin (Life Technologies), 270 unit/ml DNase (Life Technologies), and antibiotics (100 U/l penicillin and 100 μg/ml streptomycin). After gentle rotation overnight at 4 °C, tissues were washed thoroughly with PBS and the tissues were then cultured in a complete DMEM-F12 culture medium containing 10% MSC Certified fetal bovine serum (MSC-FBS; Life Technologies), 100 μg/ml of l-glutamate, and antibiotics (100 U/l penicillin and 100 μg/ml streptomycin). Tissues were then incubated at 37 °C in a humidified atmosphere containing 5% CO_2_, within a cell culture incubator. When cells migrated out of the explants, they were harvested with TrypLE™ Express detachment solution (Life Technologies) and then characterized by flow cytometry using well-characterized MSCs and haematopoietic markers (Table 1). The MSC differentiation potential into adipocytes, chondrocytes, and osteocytes was evaluated as published previously [11]. pMSCs (passage 2) from a total of 20 placentae were used in this study.

**Table 1:** Antibodies used in this study

MSC positive marker	Haematopoietic marker
CD44	CD14
CD90	CD19
CD105	CD40
CD146	CD45
CD166	CD80
HLA-ABC	CD83
	CD86
	HLA-DR

*MSC* mesenchymal stem cell

### Isolation and culture of human umbilical vein endothelial cells

Endothelial cells from human umbilical cord veins (HUVECs) were isolated according to our published method [15]. Briefly, the cannulated umbilical vein was rinsed with sterile PBS (pH 7.4) several times, and then filled with a PBS solution containing 6 mg/ml collagenase type II (Catalog # 17101-015; Life Technologies). After 25 min of incubation at 37 °C in a cell culture incubator, HUVECs were collected, resuspended in a complete endothelial cell growth medium (Catalog # PCS-100-041™; ATCC, USA), and then cultured at 37 °C in a cell culture incubator. Before using HUVECs in subsequent experiments, they were characterized by flow cytometry using a CD31 endothelial cell marker (R & D Systems, Abingdon, UK). HUVECs (> 95% purity) from passages 3–5 of a total of 30 umbilical cords were used in this study.

### Cell proliferation in response to glucose

Cells (pMSCs and HUVECs) at a density of 5 × 10^3^ were seeded in wells of 96-well culture plates containing a complete cell culture growth medium (i.e. complete DMEMF-12 culture medium for pMSCs, and complete endothelial cell growth medium for HUVECs) and then incubated at 37 °C in a cell culture incubator. At 75% confluency, non-adherent pMSCs or HUVECs were removed and cells were cultured in a complete cell culture growth medium with or without glucose (Prince Care Pharma Pvt. Ltd, India), and then incubated at 37 °C in a cell culture incubator. Different concentrations of glucose (0–2000 mM) and various culture time points (i.e. 24, 48, and 72 h) were examined. The viability of pMSCs and HUVECs was determined by the Trypan blue exclusion assay.

The proliferation of pMSCs and HUVECs was evaluated after each indicated culture time point (i.e. 24, 48, and 72 h) by a tetrazolium compound (3-(4,5-dimethylthiazol-2-yl)-5-(3-carboxymethoxyphenyl)-2-(4-sulfophenyl)-2H-tetrazolium, inner salt (MTS)) kit (Catalog # G5421, CellTiter 96® Aqueous Non-Radioactive Cell Proliferation Assay; Promega, Germany), as described previously [14]. The blank was cells incubated in MTS solution in a complete cell culture growth medium. Results were presented as means (± standard errors). Each experiment was performed in triplicate and repeated with five independent pMSC (passage 2) and HUVEC (passage 3–5) preparations.

### HUVEC proliferation in response to glucose in presence of different treatments of pMSCs

HUVECs (5 × 10^3^ cells) were seeded in wells of 96-well culture plate containing a complete endothelial cell growth medium and cultured at 37 °C in a cell culture incubator. After 24 h, adherent HUVECs were cultured alone, or co-cultured with different concentrations (20, 50, and 100 mM) of glucose in the presence of 25% CMpMSC (conditioned medium of unstimulated pMSCs, produced as described previously [14]) and pMSCs (whole cells) at a ratio of 1 HUVEC:1 pMSC. These concentrations and ratios of CMpMSC and pMSCs, respectively, were chosen because they can induce optimum HUVEC proliferative responses as reported previously by us [14]. Cells were then cultured in a complete endothelial cell growth medium for 72 h at 37 °C in a cell culture incubator.

HUVEC proliferation was then evaluated by the MTS assay as described previously [14]. Before adding pMSCs to the HUVEC culture, pMSCs were treated with 25 μg/ml Mitomycin C to inhibit their proliferation as described previously [14]. The blank was cells incubated in MTS solution in a complete endothelial cell growth medium. Results were presented as means (± standard errors). Each experiment was performed in triplicate and repeated as already described.

### Culture of HUVECs with glucose and different treatments of pMSCs (conditioned medium and intercellular direct contact)

**Fig. 1:**
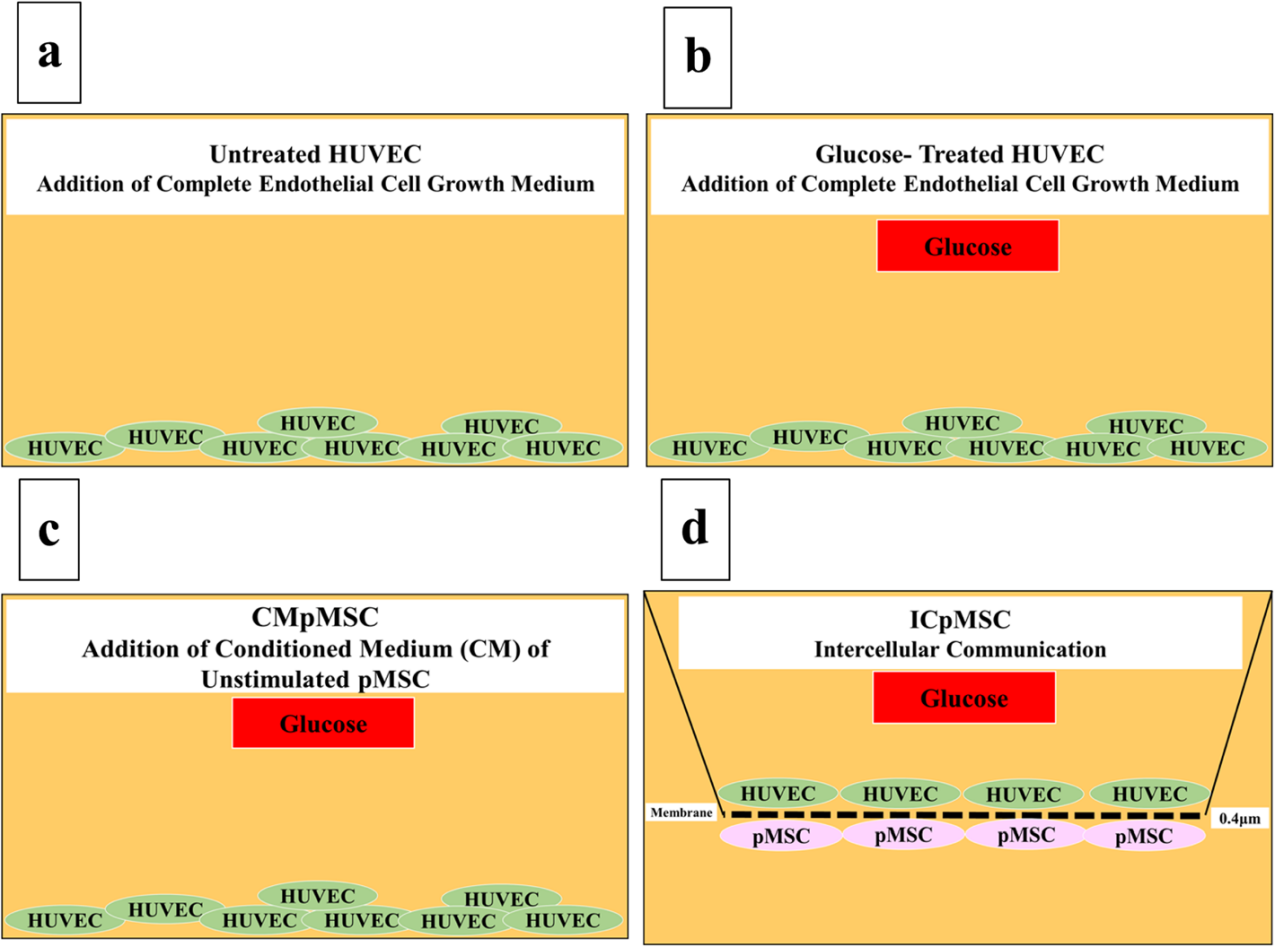
HUVEC culture system consisted of HUVECs seeded on surface of six-well culture plate in complete HUVEC culture medium alone (untreated HUVECs) (**a**), or with 100 mM glucose (**b**), or with 100 mM glucose and 25% conditioned medium (CM) obtained from unstimulated pMSCs (**c**), and ICpMSC (intercellular direct contact experiment) culture system consisted of pMSCs seeded on reverse side of membrane of chamber and HUVECs seeded on upper side of membrane (**d**). For ICpMSC, 0.4-μm pore size transwell chamber membranes were used. In ICpMSC culture system, cells cultured at a ratio of 1 HUVEC:1 pMSC in HUVEC culture medium in presence of 100 mM glucose. In all culture systems, cells incubated for 24, 48, and 72 h at 37 °C in a cell culture incubator. CMpMSC conditioned medium of unstimulated pMSCs, HUVEC human umbilical vein endothelial cell, pMSC placental mesenchymal stem cell

HUVECs were cultured alone in a complete endothelial cell growth medium (Fig. 1a), or with 100 mM glucose (Fig. 1b), or with 100 mM glucose and 25% CMpMSC (Fig. 1c). For the intercellular direct contact experiment (ICpMSC, Fig. 1d), HUVECs and pMSCs were separated by a transwell chamber membrane culture system (Catalog # 657640, ThinCert™ Cell Culture Inserts (0.4 μm); Greiner Bio-One, Germany). pMSCs were seeded on the reverse side of the membrane of the chamber while HUVECs were seeded on the upper side of the membrane as described previously [14]. Cells were cultured at a ratio of 1 HUVEC:1 pMSC in a complete endothelial cell growth medium in the presence of 100 μM glucose and incubated as already described. After 72 h, HUVEC were harvested with TrypLE™ Express detachment solution and used in the adhesion, proliferation, migration, and invasion experiments (see later). HUVEC viability was determined using the Trypan blue exclusion assay. Each experiment was performed and repeated as already described. HUVECs cultured in complete endothelial cell growth medium without glucose or pMSCs were included as negative controls for all HUVECs cultured with glucose, and different treatments of pMSCs.

### HUVEC adhesion and proliferation using xCELLigence system

The xCELLigence system (RTCA-DP version; Roche Diagnostics, Mannheim, Germany) was used as described previously [14, 15] to evaluate the adhesion and proliferation of HUVECs. The xCELLigence system is a real-time cell analyser that constantly monitors and records the changes in electrical impedance that arise from cellular events, and these changes are reported as an arbitrary cell index [14, 15]. Briefly, 100 μl complete endothelial cell growth medium was added to well in 16-well culture plates (Catalog # 05469813001, E-Plate 16; Roche Diagnostics), and the background impedance was then achieved as described previously [14, 15]. Then, 2 × 10^5^ HUVECs (initially cultured alone or co-cultured with 100 mM glucose, or with 100 mM glucose and CMpMSC, or with 100 mM glucose and ICpMSC, as already described) were seeded in 100 μl of complete endothelial cell growth medium in quadruplicate wells and equilibrium was achieved as described previously [14, 15]. For the adhesion experiments, two treatment groups of HUVECs were used. Group one consisted of HUVECs pretreated as already described, and group two consisted of HUVECs seeded in wells of the E-Plate 16 containing complete endothelial cell growth medium with 100 mM glucose or with 100 mM glucose and 25% CMpMSC.

To record data, culture plates were placed in the xCELLigence system at 37 °C in a cell culture incubator. The HUVEC cell index was then monitored in real time for 72 h. For data analysis, the xCELLigence software (version 1.2.1) was used. For cell adhesion, data were measured after 2 h, and the value of cell index was then expressed as mean ± standard error of the cell index. For cell proliferation, data were expressed as mean ± standard error of the cell index normalized to the cell index recorded after 2 h (i.e. the adhesion time point). The rate of cell proliferation was determined by calculating the normalized cell index at 24, 48, and 72 h. Each experiment was performed and repeated as already described.

### HUVEC migration using xCELLigence system

**Fig. 2:**
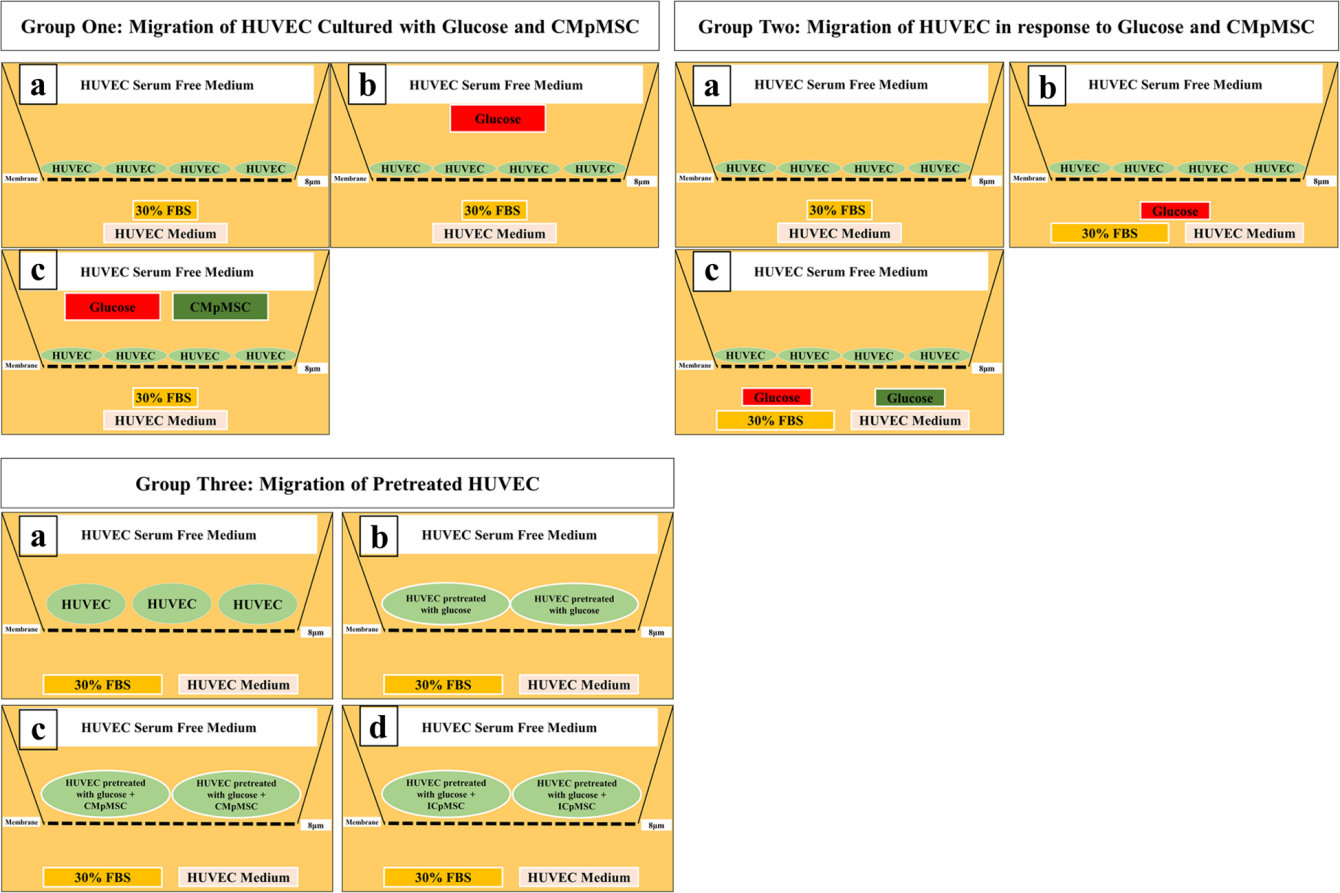
HUVEC migration groups. Group 1: HUVECs cultured alone (a), or with 100 mM glucose (b), or with 100 mM glucose and 25% conditioned medium obtained from unstimulated pMSC culture (CMpMSC) (c) in upper chamber of CIM migration plate, while HUVEC medium with 30% FBS added to lower chamber. Group 2: HUVECs seeded in HUVEC serum-free medium in upper chamber of CIM migration plate while HUVEC medium with 30% FBS (a), or with 100 mM glucose (b), or with 100 mM glucose and 20% CMpMSC (b) added to lower chamber of migration plate. Group 3: HUVEC cultured alone (a), or with 100 mM glucose (b), or with 100 mM glucose and 25% CMpMSC (c), or with pMSCs at ratio of 1 HUVEC:1 pMSC in intercellular direct contact experiment (ICpMSC) (d). Pretreated HUVECs seeded in HUVEC serum-free medium in upper chamber of CIM migration plate while HUVEC culture medium containing 30% FBS added to lower chambers. CMpMSC conditioned medium of unstimulated pMSCs, FBS fetal bovine serum, HUVEC human umbilical vein endothelial cell, pMSC placental mesenchymal stem cell

The migration of HUVECs was evaluated using CIM migration plates (Catalog # 05665825001; Roche Diagnostics) in the xCELLigence system as described previously [14, 15]. The CIM plates have 16 migration wells that each consist of two chambers (upper and lower) separated by a membrane (polyethylene terephthalate) with a pore size of 8 μm. The membrane is in contact with microelectrodes. In the migration experiments, three treatment groups were used as illustrated in Fig. 2. Group one consisted of HUVECs seeded in the upper chamber containing HUVECs in serum-free medium alone, or supplemented with 100 mM glucose, or with 100 mM glucose and 25% CMpMSC, while HUVEC medium was supplemented with 30% FBS and added to the lower chamber. Group two consisted of HUVECs seeded in the upper chamber containing HUVEC serum-free medium, while HUVEC medium supplemented with 100 mM glucose alone or with 100 mM glucose and 25% CMpMSC, and with HUVEC medium supplemented with 30% FBS, was added to the lower chamber. Group three consisted of HUVECs that were initially cultured alone, or co-cultured with 100 mM glucose, or with 100 mM glucose and CMpMSC, or with 100 mM glucose and ICpMSC as already described. HUVECs were seeded in the upper chamber in HUVEC serum-free medium while HUVEC medium supplemented with 30% FBS was added to the lower chamber. Following the addition of 50 μl pre-warmed media to the wells of the upper chamber and 160 μl endothelial cell growth medium containing 30% FBS to the lower chamber, the plates were then locked in the RTCA DP device at 37 °C in a cell culture incubator for 1 h to obtain equilibrium, and a measurement step was then performed as described previously [14, 15]. Group one measured HUVEC migration, under the effect of glucose and CMpMSC added to the upper chamber of the plate. Group two measured HUVEC migration in response to glucose and CMpMSC added to the lower chamber of the plate. Group three measured HUVEC migration after cell exposure to glucose and different treatments of pMSCs. To initiate the experiment, HUVECs (above) were seeded at a density of 2 × 10^4^ cells in the upper chamber in 100 μl, and the plates were then incubated for 30 min at RT to allow the cells to settle onto the membrane as described previously [14, 15]. Each condition was performed in quadruplicate, and after equilibration the analyser was programmed to measure electrical impedance every 15 min for 24 h. The impedance value of each well was automatically monitored by the xCELLigence system for a duration of 24 h and expressed as a CI value. Migration observed in the presence of 30% FBS, and with medium alone, served as positive and negative controls, respectively. Each experiment was performed and repeated as already described.

### pMSC effect on monocyte invasion of endothelial cell monolayer

To evaluate the permeability of HUVECs, the ability of monocytes (THP-1) to invade a monolayer of HUVECs was evaluated using the E-Plate 16 and the xCELLigence system. In the invasion experiments, two treatment groups were used. Group one consisted of HUVECs seeded in wells of the E-Plate 16 containing complete endothelial cell growth medium with 100 mM glucose alone, or with 100 mM glucose and 25% CMpMSC. Group two consisted of HUVECs initially cultured alone, or co-cultured with 100 mM glucose alone, or with 100 mM glucose and CMpMSC, or with 100 mM glucose and ICpMSC. To initiate the invasion experiments, 2 × 10^4^ HUVECs were seeded in a 16-well culture E-Plate as already described. When cells reached a growth plateau, monocytes (10^4^ cells) were added to the HUVEC monolayer. Data for cell invasion were measured and expressed as a cell index with the value expressed as mean ± standard error of the cell index. After 10 h, the rate of cell invasion was determined by calculating the normalized cell index at the pausing time (15–20 h) of the growth of HUVECs. Five experiments were performed in triplicate using HUVECs and pMSCs as already described.

### Tube formation experiments

Aliquots (100 μl) of Matrigel® Growth Factor Reduced (GFR) Basement (Catalog # 354230; Corning, USA) were plated into individual wells of 96-well tissue culture plates (Becton Dickinson) and allowed to polymerize overnight at 37 °C in a cell culture incubator. In the tube formation experiments, three treatment groups were used. Group one consisted of HUVECs cultured alone. Group two consisted of HUVECs cultured with 100 mM glucose. Group three consisted of HUVECs cultured with 100 mM glucose and 25% CMpMSC. Group four consisted of HUVECs cultured with 100 mM glucose and pMSCs. Varying pMSC:HUVEC ratios (1:30, 1:6, and 1:4) were used.

HUVECs were seeded at a density of 3 × 10^4^ cells per well in a complete endothelial cell growth culture medium on the polymerized Matrigel. Following 14 h, the tube network formed was observed under an inverted Nikon ECLIPSE Ti U microscope (Nikon, Japan). Photomicrographs were recorded using a Nikon DS-Qi1 camera and data were analysed with the software (Nikon, Japan). Experiments were carried out in triplicate and repeated as already described.

### RNA isolation, cDNA synthesis, and real-time polymerase chain reaction analysis

The expression of 84 genes related to endothelial cell biology (Catalog # PAHS-015ZD-24; Qiagen, Hilden, Germany) by HUVECs was determined using QuantiTect Primer Assay (Qiagen) in a real-time polymerase chain reaction (RT-PCR) as published previously [16]. Briefly, total RNA extracted from HUVECs initially co-cultured with 100 mM glucose alone or in the presence of different treatments of pMSCs (CMpMSC and ICpMSC) for 72 h was isolated. cDNA was then synthesized and the real-time PCR reaction was performed in triplicate on the CFX96 real-time PCR detection system (BIO-RAD) as published previously [16]. To analyse the data, the CFX manager software (Bio-Rad, CA, USA) was used. The results were exported to Microsoft Excel for further analysis. The results were expressed as the fold change by calculating the ΔΔ^− 2^ values. The relative expression of an internal house-keeping gene as a loading control was used as provided in the kit. Experiments were performed in triplicate and repeated three times using HUVECs and pMSCs as already indicated.

### Flow cytometry

Cells were characterized by flow cytometry as described previously [14]. Briefly, cells (1 × 10^5^) were stained with monoclonal antibodies (Table 1) for 30 min. Cells were then washed twice by adding cold PBS and centrifuged at 150 × *g* for 5 min at 8 °C. Unstained and isotype controls were used. Immunoreactivity to cell surface antibody markers or intracellular proteins was assayed by a BD FACS CANTO II (Becton Dickinson, NJ, USA) flow cytometer.

### Statistical analysis

Data were analysed using an unpaired *t* test, two tailed. These analyses were performed using GraphPad Prism 5. Results were considered to be statistically significant if *p* < 0.05.

## Results

### Isolation and characterization of pMSCs

MSCs from the chorionic villi of human term placentae (pMSCs) were isolated and characterized using our published methodologies [11]. pMSCs at passage 2 were positive (> 95%) for MSC markers, were negative for haematopoietic markers, and were able to differentiate into adipocytes, chondrocytes, and osteocytes. These characteristics of pMSCs were consistent with our previous report [11]. Subsequently, pMSCs at passage 2 were used in all experiments.

### Glucose effects on the proliferative potential of pMSCs and HUVECs

**Fig. 3:**
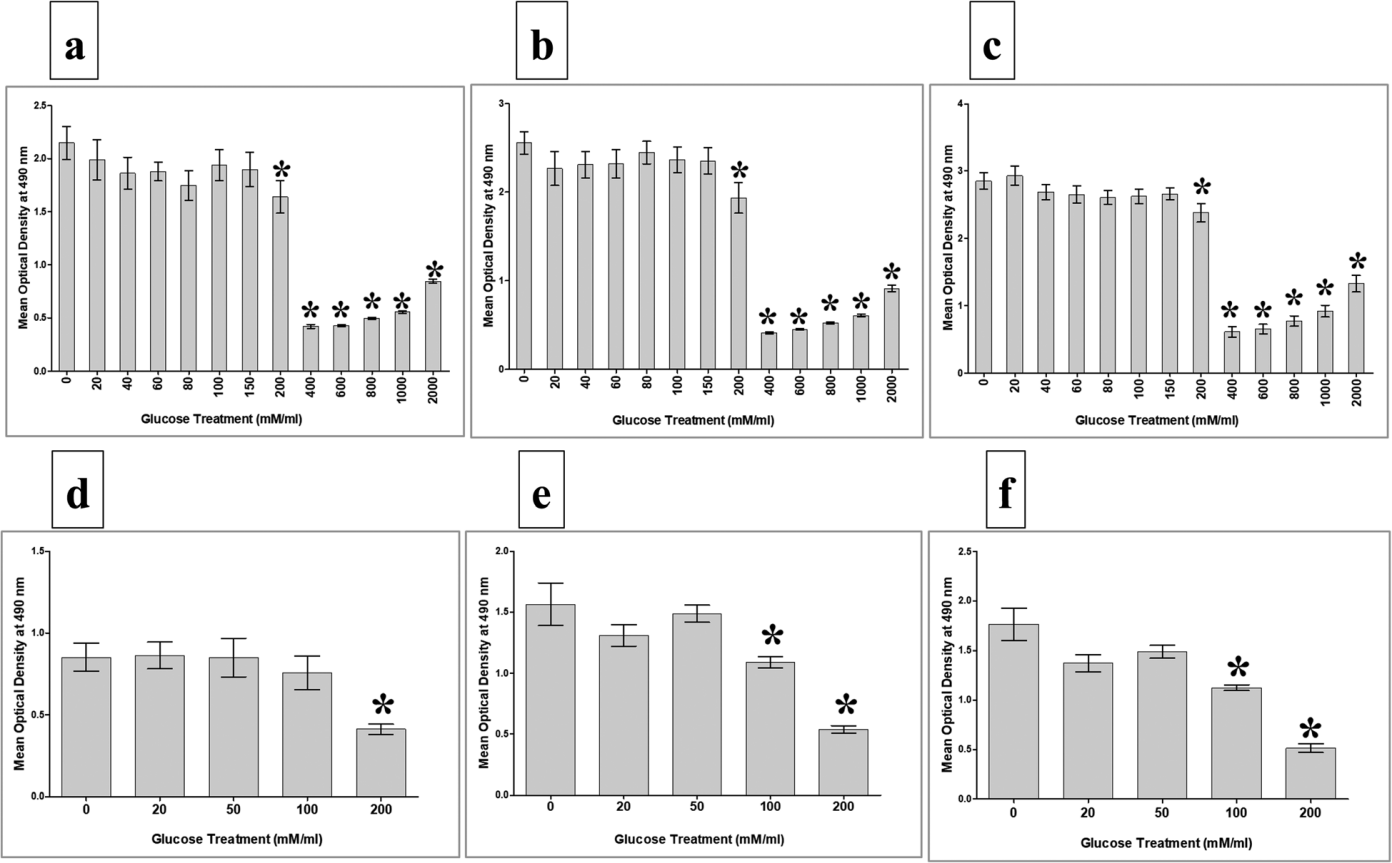
Proliferation of pMSCs and HUVECs in response to various concentrations of glucose. MTS proliferation assay showed proliferation of pMSCs in response to 20, 40, 60, 80, 100, and 150 mM glucose did not significantly change as compared to glucose-untreated pMSCs after 24 h (**a**), 48h (**b**), and 72h (**c**) while treatment with 200–2000 mM glucose for 24 h (**a**), 48 h (**b**), and 72 h (**c**) significantly decreased proliferation of pMSCs as compared to glucose-untreated pMSCs. HUVEC proliferation in response to 20 and 50 mM glucose did not significantly change as compared to glucose-untreated HUVECs after 24 h (**d**), 48 h (**e**), and 72 h (**f**). Culture of HUVECs with 100 mM glucose for 24 h (**d**) did not significantly change proliferation, but significantly decreased proliferation after 48h (**e**) and 72 h (**f**) as compared to glucose-untreated pMSCs. HUVEC proliferation in response to 200 mM glucose significantly reduced as compared to glucose-untreated HUVECs after 24 h (**d**), 48 h (**e**), and 72 h (**f**). Each experiment performed in triplicate using pMSCs (passage 2) and HUVECs (passage 3–5) from five independent placentae and umbilical cord tissues. *p < 0.05. Bars represent standard errors

To evaluate the proliferative response of pMSCs to glucose, pMSCs were cultured alone, or with glucose, and the proliferation potential was then determined using the MTS assay. After 24, 48, and 72 h treatment with 200–2000 mM glucose, pMSC proliferation was significantly reduced (*p* > 0.05) as compared to glucose-untreated pMSCs (Fig. 3a–c), while the treatment with 20–150 mM glucose had no significant effect (*p* > 0.05) on pMSC proliferation at all culture time points (24–72 h) (Fig. 3a–c). The viability of pMSCs treated with 20–200 mM glucose was > 95% while the exposure of pMSCs to a concentration of glucose higher than 200 mM significantly reduced cell viability (< 50%) for all culture time points.

Next, we evaluated the proliferation of HUVECs in response to 20–2000 mM glucose. HUVEC proliferation did not significantly change (*p* > 0.05) after treating the cells with 20 and 50 mM glucose as compared to glucose-untreated HUVECs for all culture time points (Fig. 3d–f), while the treatment with 100 mM glucose significantly reduced HUVEC proliferation (*p* < 0.05) as compared to glucose-untreated HUVECs only after 48 and 72 h (Fig. 3e, f), but not after 24 h (*p* > 0.05, Fig. 3d,). In contrast, the treatment with 200 mM glucose significantly reduced HUVEC proliferation as compared to glucose-untreated HUVECs after 24, 48, and 72 h (*p* < 0.05, Fig. 3d–f). The viability of HUVECs treated with 20, 50, and 100 mM glucose was > 95% while the treatment with 200 mM glucose reduced the viability to less than 50% for all culture time points. The exposure of HUVECs to glucose concentrations higher than 200 mM significantly reduced (< 20%) the viability for all culture time points.

Based on the results obtained, the exposure time of 72 h and 100 mM glucose were selected to evaluate the effect of glucose on the functions of HUVECs (proliferation, adhesion, migration, permeability, and tube network formation).

### pMSCs and glucose modulate the proliferation of HUVECs

**Fig. 4:**
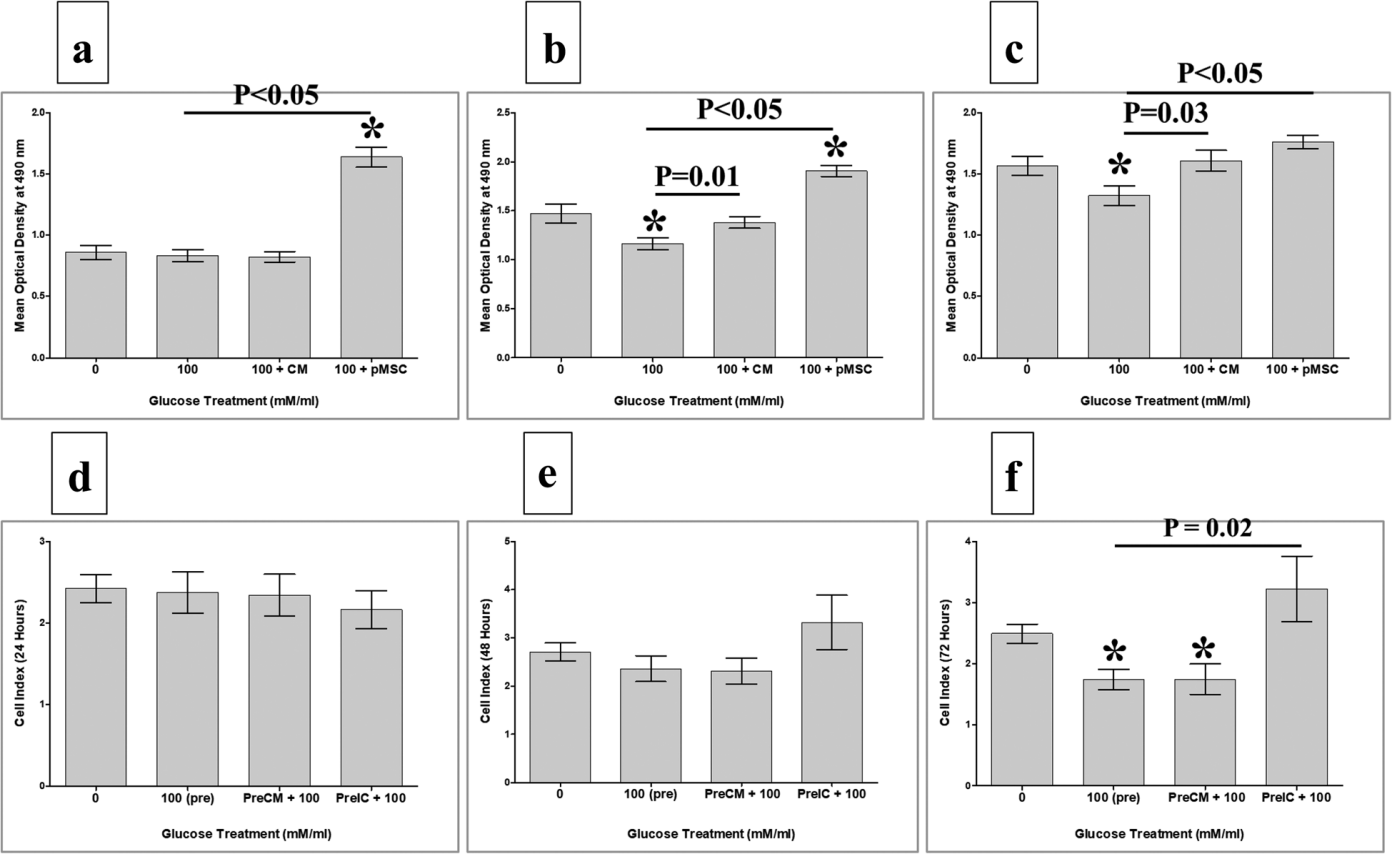
HUVEC proliferation in response to glucose in presence of pMSCs, or after removing glucose and pMSCs, examined after 24, 48, and 72 h in MTS assay. In response to conditioned medium (CMpMSC), pMSCs had no significant effect on HUVEC proliferation in presence of glucose after 24 h as compared to untreated or glucose-treated HUVECs (**a**). CMpMSC significantly increased HUVEC proliferation in presence of glucose after 48 h (**b**) and 72 h (**c**), as compared to glucose-treated but not untreated HUVECs. Cell–cell contact assay showed that, compared to glucose-untreated or treated HUVECs, pMSCs significantly increased HUVEC proliferation in presence of glucose after 24 h (**a**) and 48 h (**b**), while after 72 h (**c**) pMSCs significantly increased HUVEC proliferation in presence of glucose, as compared to glucose-treated but not untreated HUVECs. HUVEC proliferation after removing effects of glucose and pMSCs. HUVECs initially cultured with 100 mM glucose (100(pre)) in presence of different treatments of pMSCs (CMpMSC(PreCM + 100) and ICpMSC(PreCM + 100)) for 72 h, and then used in proliferation assay using xCELLigence real-time cell analyser. After 24 and 48 h (**d, e**), proliferation of HUVECs pretreated with glucose alone (100(pre)), or with CMpMSC (PreCM + 100) or ICpMSC (PreCM + 100), did not significantly change as compared to glucose-untreated HUVEC (*p* > 0.05). As compared to glucose-treated HUVECs (100(pre)), proliferation of HUVECs pretreated with glucose and CMpMSC (PreCM + 100), or glucose and ICpMSC (PreIC + 100), did not significantly change after 24 and 48 h (*p* > 0.05) (**d, e**). In contrast, proliferation of HUVECs pretreated with 100 mM glucose alone (100(pre)), or with CMpMSC (PreCM + 100), significantly reduced after 72 h as compared to glucose-untreated HUVEC (**f**). When compared with glucose-treated HUVECs (100(pre)), proliferation of HUVECs pretreated with glucose and CMpMSC (PreCM + 100) did not significantly change after 72 h of culture. In contrast, proliferation of HUVECs pretreated with glucose and ICpMSC (PreIC + 100) increased significantly after 72 h of culture as compared to glucose-treated but not untreated HUVECs (**f**). Each experiment performed in triplicate using HUVECs (passage 3–5) and pMSCs (passage 2) from five independent umbilical cord tissues and placentae, respectively. *P value is significant *p* < 0.05. Bars represent standard errors. pMSC placental mesenchymal stem cell

To evaluate the effects of pMSCs on endothelial cell functions in response to glucose, the proliferation of HUVECs cultured with 100 mM glucose alone, or with 100 mM glucose and different treatments of pMSCs (CMpMSC and pMSCs), was examined using the MTS assay. The treatment of HUVEC with glucose, HUVEC proliferation significantly reduced after 48 and 72 h (*p* < 0.05), but not after 24 h (*p* > 0.05), as compared to glucose-treated HUVECs (Fig. 4). The addition of CMpMSC to glucose-treated HUVEC significantly induced the proliferation of HUVECs after 48 and 72 h (*p* < 0.05), but not after 24 h, as compared to glucose-treated HUVECs (Fig. 4a–c). However, the addition of pMSCs to glucose-treated HUVECs significantly induced the proliferation of HUVECs after 24, 48, and 72 h (*p* < 0.05) as compared to glucose-treated HUVECs (Fig. 4a–c). The addition of glucose to HUVECs in the presence of CMpMSC had no significant effect on HUVEC proliferation (*p* > 0.05) as compared to glucose-untreated HUVECs after all culture time points, while the addition of glucose to HUVECs in the presence of pMSCs significantly increased the proliferation of HUVECs after 24 and 48 h (*p* < 0.05), but not after 72 h (*p* > 0.05), as compared to glucose-untreated HUVECs (Fig. 4a–c).

### The reversibility of HUVEC proliferation in response to glucose and pMSCs

To evaluate the reversibility effect of pMSCs on the proliferation of glucose-treated HUVECs, HUVECs were initially cultured alone, or with 100 mM glucose alone, or with 100 mM glucose and different treatments of pMSCs (CMpMSC and ICpMSC) for 72 h and their proliferation was measured using the xCELLigence system. After 24 and 48 h, the proliferation of glucose-pretreated HUVECs (100(Pre)) was not significantly changed (*p* > 0.05), but after 72 h it was significantly reduced (*p* < 0.05) as compared to glucose-untreated HUVECs (Fig. 4f). These data show that glucose has an irreversible inhibitory effect on HUVEC proliferation. The proliferation of HUVECs pretreated with glucose and CMpMSC (PreCM + 100) did not significantly change (*p* > 0.05) after 24 and 48 h, as compared to glucose-untreated HUVECs or glucose-pretreated HUVECs (100(Pre)) (Fig. 4d, e). However, proliferation was significantly reduced (*p* < 0.05) or unchanged (*p* > 0.05) after 72 h as compared to untreated HUVECs and glucose-pretreated HUVECs (100(Pre)), respectively (Fig. 4f). Therefore, the stimulatory effect of CMpMSC on glucose inhibiting HUVEC proliferation is reversible. The proliferation of HUVECs pretreated with glucose and ICpMSC (PreIC + 100) did not significantly change (*p* > 0.05) after 24 and 48 h, as compared to glucose-untreated HUVECs and glucose-pretreated HUVECs (100(Pre)) (Fig. 4d, e). However, proliferation significantly increased (*p* < 0.05) or was unchanged (*p* > 0.05) after 72 h as compared to glucose-pretreated HUVECs (100(Pre)) and glucose-untreated HUVECs, respectively (Fig. 4f). These data show that the stimulatory effect of ICpMSC on glucose inhibition of HUVEC proliferation is irreversible.

### pMSCs and glucose effects on HUVEC adhesion

**Fig. 5:**
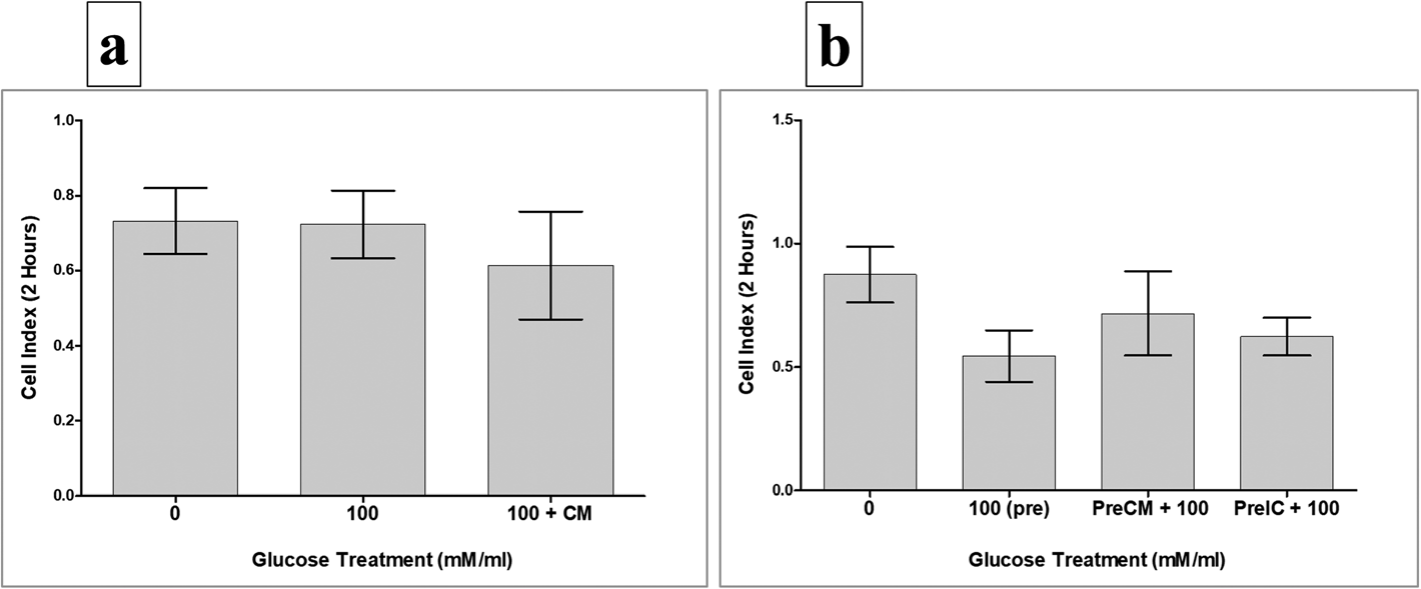
HUVEC adhesion in response to glucose and pMSCs, or after removing effects of glucose and pMSCs. HUVECs cultured with 100 mM glucose alone (100), or with 25% CMpMSC (100 + CM), and adhesion then measured using xCELLigence real-time cell analyser. After 2 h, as compared to glucose-untreated HUVECs, HUVEC adhesion in presence of glucose alone (100), or with CMpMSC (100 + CM), did not significantly change (*p* > 0.05) (**a**). Adhesion of HUVECs in presence of glucose and CMpMSC (100 + CM) did not significantly change as compared to glucose-treated HUVECs (100) after 2 h (*p* > 0.05) (**a**). HUVECs pretreated with 100 mM glucose (100(pre)) in presence of different pMSC treatments (CMpMSC (PreCM + 100) and ICpMSC (PreIC + 100)) were cultured in 16-well culture plate and adhesion measured as already indicated. After 2 h, and as compared to glucose-untreated HUVECs, preteatment of HUVECs with glucose (100(pre)), or with glucose and CMpMSC (PreCM + 100), or with glucose and ICpMSC (PreIC + 100), did not significantly change (*p* > 0.05) (**b**). Adhesion of HUVECs in presence of glucose and CMpMSC (PreCM + 100), or glucose and ICpMSC (PreIC + 100), did not significantly change as compared to glucose-treated HUVECs (100(pre)) after 2 h (*p* > 0.05) (**b**). Each experiment performed in triplicate using HUVECs (passage 3–5) and pMSCs (passage 2) from five independent umbilical cord tissues and placentae, respectively. Bars represent standard errors

To study the effects of pMSCs and glucose on the adhesion of HUVECs, two HUVEC treatment groups were evaluated as already described. After 2 h, the adhesion of HUVECs treated with 100 mM glucose alone (100), or with 100 mM glucose and CMpMSC (100 + CM), did not significantly change as compared to glucose-untreated HUVECs (*p* > 0.05, Fig. 5a). Similarly, after 2 h, the adhesion of HUVECs pretreated with 100 mM glucose alone (100(Pre)), or with 100 mM glucose and CMpMSC (PreCM + 100), or 100 mM glucose and ICpMSC (PreIC + 100), were reduced but not statistically significant as compared to glucose-untreated HUVECs (*p* > 0.05, Fig. 5b). In addition, the pretreatment of HUVECs with 100 mM glucose and CMpMSC (PreCM + 100), or with 100 mM glucose and ICpMSC (PreIC + 100), did not affect the adhesion of HUVECs as compared to glucose-pretreated HUVECs (100(Pre)) (*p* > 0.05, Fig. 5b).

### pMSCs and glucose modulate HUVEC migration

**Fig. 6:**
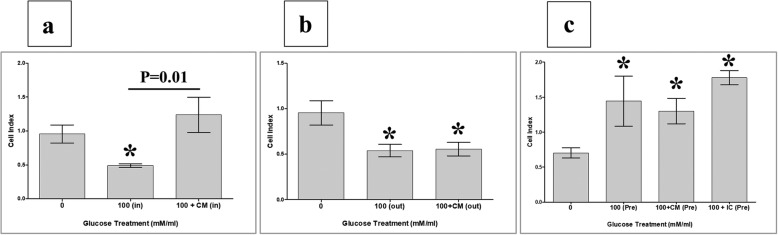
HUVEC migration measured using xCELLigence real-time cell analyser. After 24 h, migration of HUVECs cultured with 100 mM glucose (100(in)) significantly reduced as compared to glucose-untreated HUVECs (**a**). As compared to glucose-untreated HUVECs, HUVEC migration in presence of glucose and CMpMSC (100 + CM(in)) did not significantly change (*p* > 0.05) after 24 h but migration significantly increased as compared to glucose-treated HUVECs (100(in)) (**a**). Migration of HUVECs in response to 100 mM glucose alone (100(out)), or with CMpMSC (100 + CM(out)) added to lower chamber of migration plate, significantly reduced as compared to glucose-untreated HUVECs after 24 h (**b**). As compared to glucose-treated HUVECs (100(out)), HUVEC migration in response to glucose and CMpMSC (100 + CM(out)) did not significantly change after 24 h as compared to glucose-treated HUVECs (100(out)) (**b**). After 24 h, migration of HUVECs pretreated with glucose alone (100(pre), or with glucose and CMpMSC (100 + CM(Pre)), or with glucose and ICpMSC (100 + IC(Pre)), significantly increased as compared to glucose-treated HUVECs (100(pre)) (**c**). After 24 h, as compared to glucose-treated HUVECs (100(pre)), migration of HUVECs pretreated with 100 mM glucose and CMpMSC (100 + CM(Pre)), or with 100 mM glucose and ICpMSC (100 + IC(Pre)), did not significantly change (*p* > 0.05) (**c**). Each experiment performed in triplicate using HUVECs (passage 3–5) and pMSCs (passage 2) from five independent umbilical cord tissues and placentae, respectively. *P value is significant < 0.05. Bars represent standard errors

We further evaluated the migration of HUVECs exposed to 100 mM glucose alone (100(in)) or to 100 mM glucose and CMpMSC (100 + CM(in)) during the migration assay (migration group one, Fig. 2). After 24 h incubation with glucose (100(in)), HUVEC migration significantly reduced (*p* < 0.05) as compared to glucose-untreated HUVECs while the incubation with glucose and CMpMSC (100 + CM(in)) had no significant effect (*p* > 0.05) on HUVEC migration as compared to glucose-untreated HUVECs (Fig. 6a). As compared to glucose-treated HUVECs (100(in)), HUVEC migration in the presence of glucose and CMpMSC (100 + CM(in)) significantly increased (*p* < 0.05, Fig. 6a).

We also examined the migration of HUVECs in response to 100 mM glucose alone (100(out)) or to 100 mM glucose and CMpMSC (100 + CM(out)) (migration group two, Fig. 2). After 24 h, HUVEC migration in response to glucose alone (100(out)) or to glucose and CMpMSC (100 + CM(out)) significantly reduced (*p* < 0.05) as compared to glucose-untreated HUVECs (Fig. 6b). In contrast, the migration of HUVECs in response to glucose and CMpMSC (100 + CM(out)) did not change (*p* < 0.05) as compared to glucose-treated HUVECs (100(out)) (Fig. 6b).

Next, we evaluated the effect of pretreatment with glucose and pMSCs as described in migration group three (Fig. 2). After 24 h, the migration of HUVECs pretreated with 100 mM glucose alone (100(Pre)), or with 100 mM glucose and CMpMSC (100 + CM(Pre)), or with 100 mM glucose and ICpMSC (100 + IC(Pre)), significantly increased as compared to glucose-untreated HUVECs (Fig. 6c). In comparison with glucose-pretreated HUVECs (100(Pre)), the migration of HUVECs pretreated with glucose and CMpMSC (100 + CM(Pre)), or with glucose and ICpMSC (100 + IC(Pre)), was not significantly changed (*p* < 0.05) after 24 h (Fig. 6c).

### pMSCs reduce the effect of glucose on HUVEC permeability

**Fig. 7:**
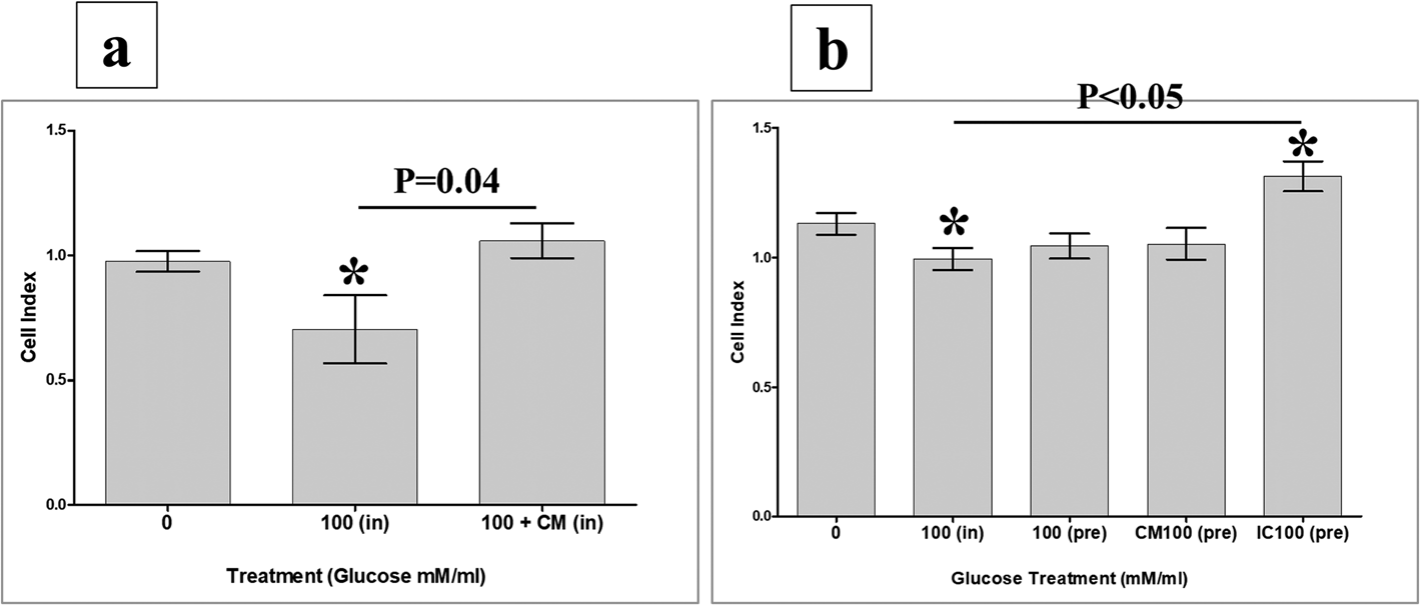
HUVEC permeability under effects of glucose and pMSCs examined by adding monocytes to monolayer of HUVECs and assessing invasion of monocytes through HUVEC monolayer by xCELLigence real-time system. Increased invasion defined as reduction in cell index due to infiltration of HUVEC monolayer by monocytes, causing detachment of HUVECs, while increased cell index defines reduction in cell invasion. In presence of 100 mM/ml glucose (100(in)), monocyte invasion of HUVEC monolayer significantly increased after 10 h as compared to glucose-untreated HUVECs (**a**). After 10 h and as compared to glucose-untreated HUVECs, monocyte invasion in presence of glucose and CMpMSC (100 + CM(in)) significantly reduced but not significantly changed as compared to glucose-untreated HUVECs (**a**). Monocyte invasion through monolayer of HUVECs pretreated with glucose alone (100(pre)), or with glucose and CMpMSC (100 + CM(pre)), not significantly changed after 10 h as compared to glucose-untreated HUVECs, while addition of ICpMSC (100 + IC(pre)) significantly reduced monocyte invasion as compared to glucose-treated (100(pre)) or untreated HUVECs (**b**). Each experiment performed in triplicate using HUVECs (passage 3–5) and pMSCs (passage 2) from five independent umbilical cord tissues and placentae, respectively. *P value is significant < 0.05. Bars represent standard errors

In the xCELLigence real-time system, increased invasion is defined as a reduction in the cell index due to the infiltration of the HUVEC monolayer by monocytes, causing detachment of HUVECs, while an increased cell index defines the reduction in cell invasion. The effect of glucose on the permeability of HUVECs in the presence or absence of CMpMSC was examined by adding monocytes to a monolayer of HUVECs and the invasion of monocytes through the HUVEC monolayer was then assessed by the xCELLigence real-time system. After 10 h and in the presence of glucose (100(in)), monocyte invasion of the HUVEC monolayer significantly increased (*p* < 0.05) as compared to glucose-untreated HUVECs (Fig. 7a). The addition of CMpMSC to glucose-cultured HUVECs (100 + CM(in)) significantly reduced (*p* < 0.05) monocyte invasion as compared to glucose-treated HUVECs (100(in)), but was not significantly changed (*p* > 0.05) as compared to glucose-untreated HUVECs (Fig. 7a).

We also evaluated the reversibility of monocyte invasion through the HUVEC monolayer by using HUVECs that were initially cultured alone, or with 100 mM glucose alone, or with 100 mM glucose and different treatments of pMSCs (CMpMSC and ICpMSC). The invasion of monocytes through the monolayer of HUVECs pretreated with glucose alone (100(pre)), or with CMpMSC (CM100(pre)), was not significantly changed as compared to glucose-untreated HUVECs, while the addition of ICpMSC (IC100(pre)) significantly reduced (*p* < 0.05) monocyte invasion as compared to glucose-treated HUVECs and untreated HUVECs (Fig. 7b).

### The effect of pMSCs on glucose inhibition of HUVEC tubule network formation in vitro

**Fig. 8:**
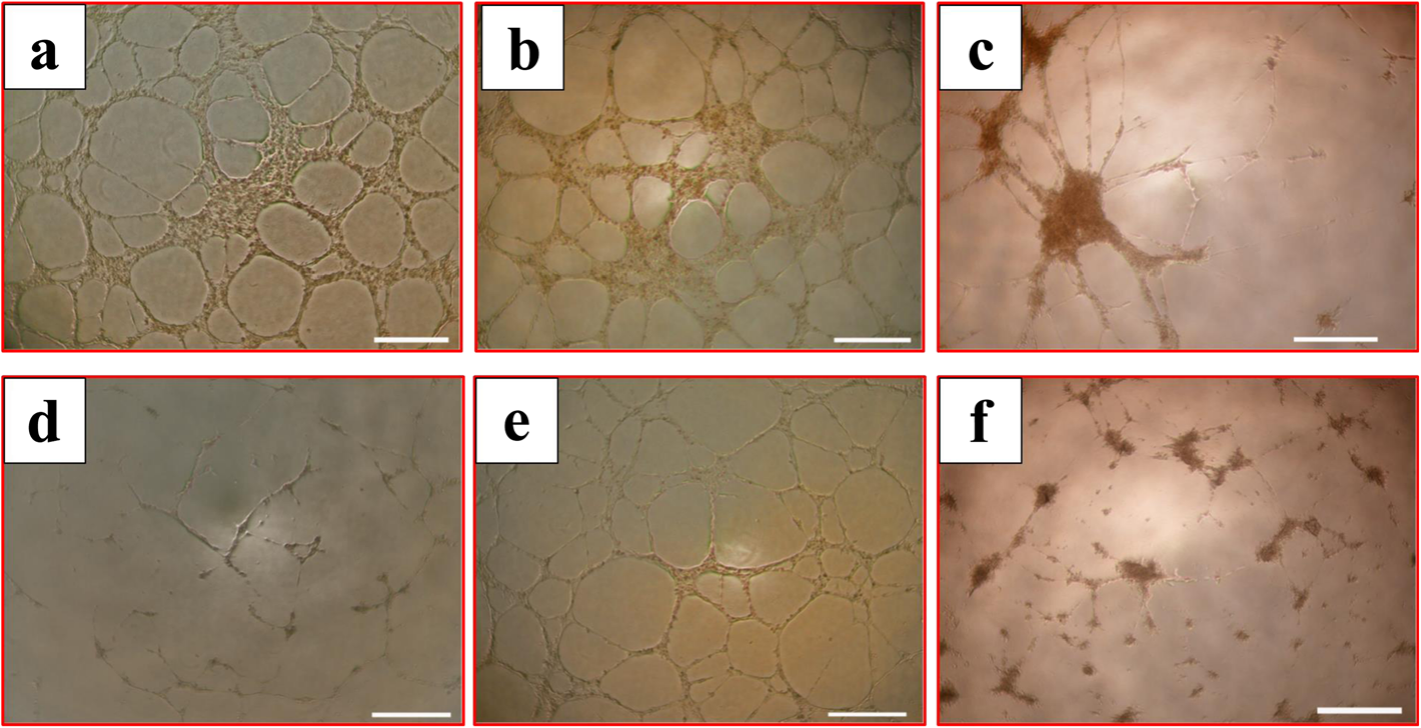
HUVEC tubule formation in presence of glucose and pMSCs. After 14 h, glucose-untreated pMSCs (**a**) and HUVECs cultured with 25% CMpMSC (**b**) able to form tube networks. HUVECs cultured with pMSCs alone did not form extensive tubule networks (**c**), and with 100 mM glucose alone (**d**) were unable to form tube networks. HUVECs cultured with 100 mM glucose and 25% CMpMSC (**e**) able to form tube networks, while culturing HUVECs with 100 mM glucose and pMSCs (**f**) failed to form extensive tube networks. Each experiment performed in triplicate using HUVECs (passage 3–5) and pMSCs (passage 2) from five independent umbilical cord tissues and placentae, respectively

To evaluate the effect of glucose on the ability of HUVECs to form tubule networks in vitro, HUVECs were seeded alone, or with 100 mM glucose, or with 100 mM glucose and different treatments of pMSCs (CMpMSC and pMSC) on a Matrigel-coated surface. After 14 h, untreated HUVECs formed tube networks (Fig. 8a). The addition of CMpMSC to the HUVEC culture had no apparent effect on tubule network formation by HUVECs (Fig. 8b). However, the co-culture of HUVECs with pMSCs resulted in limited tubule network formation by HUVECs (Fig. 8c). The incubation of HUVECs with glucose had the most dramatic effect in that it completely inhibited HUVEC tubule network formation (Fig. 8d). The addition of CMpMSC to glucose-treated HUVECs reversed the inhibitory effect of glucose on HUVEC tubule network formation (Fig. 8e). Finally, the co-culture of HUVECs with pMSCs and glucose, HUVECs were unable to form extensive tubule networks (Fig. 8f).

### pMSCs modulate the effect of glucose on the expression of genes important in endothelial cell functions

The expression of genes mediating endothelial cell functions was studied after culturing endothelial cells with glucose in the presence or absence of pMSCs for 72 h, and then analysed and assessed using the real-time PCR assay. Results show that pMSCs modulated glucose effects on endothelial cell expression of genes underlying many of the endothelial cell functional activities, including survival, apoptosis, injury, inflammation, angiogenesis, permeability, and leukocyte infiltration, as compared to glucose-treated endothelial cells (Tables 2, 3, 4, 5, 6, and 7).

**Table 2:** pMSCs modulate expression of genes involved in endothelial cell (EC) survival, apoptosis, injury, and inflammation

Number	Gene symbol	Gene full name	Glucose mean ΔΔ^−2^ value	Glucose + CMpMSC mean ΔΔ^− 2^ value	Fold change, glucose vs glucose + CMpMSC (*p* < 0.05)	Biological activity
1	*CAV1*	Caveolin-1	6	3	2-fold ↓	Inhibits EC proliferation
2	*COL18A1*	Collagen, type XVIII, alpha 1	463	4	115-fold ↓	
3	*PROCR*	Protein C receptor, endothelial	0.61	9.11	14.93-fold ↑	Induces EC survival
4	*F2R*	Protease-activated receptor-1	1.8	104.45	58.02-fold ↑	
5	*EDN1*	Endothelin-1	1	6	6-fold ↑	
6	*TYMP*	Thymidine phosphorylase	5	219	43.80-fold ↑	
7	*ENG*	Endoglin	60.47	1.79	33.78-fold ↓	Induces EC injury
8	*CX3CL1*	Chemokine ligand 1	6.85	2.55	2.68-fold ↓	
9	*F3*	Coagulation factor III, tissue factor	7.17	1.39	5.15-fold ↓	
10	*THBD*	Thrombomodulin	131.78	8.52	15.46-fold ↓	
11	*IL3*	Interleukin 3	47.75	0.0006	79,583.3-fold ↓	Induces EC inflammation
12	*ALOX5*	Arachidonate 5-lipoxygenase	103.45	17.62	53.05-fold ↓	
13	*FLT1*	Vascular endothelial growth factor receptor 1	7.14	2.75	2.59-fold ↓	

*CMpMSC* conditioned medium of unstimulated pMSCs, *pMSC* placental mesenchymal stem cell, ↓ decrease, ↑ increase

**Table 3:** pMSCs modulate expression of genes mediating endothelial cell (EC) angiogenesis

Number	Gene symbol	Gene full name	Glucose ΔΔ^−2^ value	Glucose + CMpMSC ΔΔ^− 2^ value	Fold change, glucose vs glucose + CMpMSC (*p* < 0.05)	Biological activity
1	*AGT*	Angiotensinogen	3.3	0.004	825-fold ↓	Inhibits EC angiogenesis
2	*COL18A1*	Collagen, type XVIII, alpha 1	463	4	115-fold ↓	
3	*F2R*	Protease-activated receptor-1	1.8	104.45	58.02-fold ↑	Induces EC angiogenesis
4	*ICAM1*	Intercellular adhesion molecule 1	11.5	23.85	2.07-fold ↑	
5	*PGF*	Placental growth factor	3.21	22.27	6.93-fold ↑	
6	*CCL2*	Monocyte chemotactic protein-1 (MCP-1)	17.72	35.67	2.01-fold ↑	
7	*EDN1*	Endothelin-1	1	6	6-fold ↑	Induces EC migration
8	*PF4*	Platelet factor 4	0.79	3.94	4.98-fold ↑	
9	*ICAM1*	Intercellular adhesion molecule 1	11.5	23.85	2.07-fold ↑	

*CMpMSC* conditioned medium of unstimulated pMSCs, *pMSC* placental mesenchymal stem cell, ↓ decrease, ↑ increase

**Table 4:** pMSCs modulate expression of genes mediating endothelial cell (EC) permeability and leukocyte infiltration of ECs

Number	Gene symbol	Gene full name	Glucose ΔΔ^− 2^ value	Glucose + CMpMSC ΔΔ^− 2^ value	Fold change, glucose vs glucose + CMpMSC *(p* < 0.05)	Biological activity
1	*ALOX5*	Arachidonate 5-lipoxygenase	103.45	17.62	53.05-fold ↓	Induces EC permeability
2	*ICAM1*	Intercellular adhesion molecule 1	11.5	23.85	2.07-fold ↑	
3	*NPR1*	Atrionatriuretic peptide receptor A	1.16	3.79	3.26-fold ↑	Inhibits EC permeability
4	*CAV1*	Caveolin-1	5.87	3.16	1.85-fold ↓	
5	*ENG*	Endoglin	60.47	1.79	33.78-fold ↓	Induces leukocyte infiltration
6	*VCAM1*	Vascular cell adhesion molecule 1	64.03	1.79	35.77-fold ↓	
7	*SELL*	Selectin L	22.05	35.91	1.62-fold ↑	
8	*SELE*	E-selectin	22.05	35.91	1.62-fold ↑	

*CMpMSC* conditioned medium of unstimulated pMSCs, *pMSC* placental mesenchymal stem cell, ↓ decrease, ↑ increase

**Table 5:** pMSCs modulate expression of genes involved in endothelial cell (EC) survival, apoptosis, injury, fibrosis formation, and inflammation

Number	Gene symbol	Gene full name	Glucose ΔΔ^−2^ value	Glucose + ICpMSC ΔΔ^− 2^ value	Fold change, glucose vs. glucose + ICpMSC (*p* < 0.05)	Biological activity
1	*PLAT*	Plasminogen activator, tissue	4.52	247.12	54.67-fold ↑	Induces EC proliferation
2	*PDGFRA*	Platelet-derived growth factor receptor, alpha polypeptide	38	76	2-fold ↑	
3	*CAV1*	Caveolin-1	6	2	3-fold ↓	Inhibits EC proliferation
4	*COL18A1*	Collagen, type XVIII, alpha 1	463	19	24-fold ↓	
5	*PROCR*	Protein C receptor, endothelial	0.61	7.69	12.6-fold ↑	Induces EC survival
6	*F2R*	Protease-activated receptor-1	1.8	10.91	6.06-fold ↑	
7	*TGFB1*	Transforming growth factor beta 1	0.96	37.26	138-fold ↑	
8	*BCL2L1*	BCL2-like 1	1.4	140.43	100.3-fold ↑	
9	*MMP1*	Matrix metallopeptidase 1	1.06	24	22.64-fold ↑	
10	*KDR*	Vascular endothelial growth factor receptor 3 (VEGFR3)	0.62	50.83	81.92-fold ↑	
11	*SPHK1*	Sphingosine kinase 1	1330.29	1,156,189.43	869.12-fold ↑	
12	*TNFSF10*	TNF-related apoptosis-inducing ligand (TRAIL)	1.65	1788.25	1083-fold ↑	
13	*FASLG*	Fas ligand	18,254.05	9.84	1855.08-fold ↓	Induces EC apoptosis
14	*ENG*	Endoglin	60.47	1.89	31.99-fold ↓	Induces EC injury
15	*AGTR1*	Angiotensin II receptor, type 1	2366.34	227.48	10.4-fold ↓	
16	*THBD*	Thrombomodulin	131.78	10.19	12.93-fold ↓	
17	*IL3*	Interleukin 3	47.75	0.59	80.93-fold ↓	Induces EC inflammation
18	*ALOX5*	Arachidonate 5-lipoxygenase	103.45	0.82	126.15-fold ↓	
19	*FLT1*	Vascular endothelial growth factor receptor 1	7.14	1.96	3.64-fold ↓	

*ICpMSC* intercellular direct contact experiment, *pMSC* placental mesenchymal stem cell, *TNF* tumour necrosis factor, ↓ decrease, ↑ increase

**Table 6:** pMSCs modulate expression of genes mediating endothelial cell (EC) angiogenesis

Number	Gene symbol	Gene full name	Glucose ΔΔ^− 2^ value	Glucose + ICpMSC ΔΔ^− 2^ value	Fold change, glucose vs glucose + ICpMSC (*p* < 0.05)	Biological activity
1	*TIMP1*	TIMP metallopeptidase inhibitor 1	2.28	6.7	2.93-fold ↑	Inhibits EC angiogenesis
2	*CASP1*	Apoptosis-related cysteine peptidase	0.01	8.97	897-fold ↑	
3	*PF4*	Platelet factor 4	0.79	3.54	4.48-fold ↑	
4	*AGT*	Angiotensinogen	3.3	0.54	6.1-fold ↓	
5	*COL18A1*	Collagen, type XVIII, alpha 1	463	19	24-fold ↓	
6	*F2R*	Protease-activated receptor-1	1.8	10.91	6.06-fold ↑	Induces EC angiogenesis
7	*ICAM1*	Intercellular adhesion molecule 1	11.5	642.79	55.89-fold ↑	
8	*PGF*	Placental growth factor	3.21	178.53	55.56-fold ↑	
9	*CCL2*	Monocyte chemotactic protein-1 (MCP-1)	17.72	44.51	2.52-fold ↑	
10	*PTGS2*	Cyclooxygenase (COX)	1.9	3.84	2.02-fold ↑	
11	*SPHK1*	Sphingosine kinase 1	1330.29	1,156,189.43	869-fold ↑	Induces EC migration
12	*PF4*	Platelet factor 4	0.79	3.54	4.48-fold ↑	
13	*ICAM1*	Intercellular adhesion molecule 1	11.5	642.79	55.89-fold ↑	

*ICpMSC* intercellular direct contact experiment, *pMSC* placental mesenchymal stem cell, ↓ decrease, ↑ increase

**Table 7:** pMSCs modulate expression of genes mediating endothelial cell (EC) permeability and leukocyte infiltration of ECs

Number	Gene symbol	Gene full name	Glucose ΔΔ^−2^ value	Glucose + ICpMSC ΔΔ^− 2^ value	Fold change, glucose compared with glucose + ICpMSC (*p* < 0.05)	Biological activity
1	*ALOX5*	Arachidonate 5-lipoxygenase	103.45	0.82	126.15-fold ↓	Induces EC permeability
2	*NPPB*	Natriuretic peptide B	4.12	0.04	103-fold ↓	
3	*IL1β*	Interleukin 1 beta	3.37	23.52	6.97-fold ↑	
4	*IL6*	Interleukin 6	0.43	32.23	74.95-fold ↑	
5	*ICAM1*	Intercellular adhesion molecule 1	11.5	642.79	55.89-fold ↑	
6	*CAV1*	Caveolin-1	5.87	2.5	2.34-fold ↓	Inhibits EC permeability
7	*NPR1*	Atrionatriuretic peptide receptor A	1.16	12.58	10.84-fold ↑	
1	*ENG*	Endoglin	60.47	1.89	31.99-fold ↓	Induces leukocyte infiltration
2	*VCAM1*	Vascular cell adhesion molecule 1	64.03	1.19	53.8-fold ↓	
3	*SELL*	Selectin L	22.05	359.67	16.3-fold ↑	
4	*SELE*	E-selectin	22.05	359.67	16.31-fold ↑	

* ICPMSC* intercellular direct contact experiment, *pMSC* placental mesenchymal stem cell, ↓ decrease, ↑ increase

## Discussion

In diabetes, hyperglycaemia stimulates the production of H_2_O_2_ in the endothelium that contributes to the development of endothelial injury and the development of thrombosis [2–8]. Recently, we reported the ability of pMSCs to protect endothelial cells from injury induced by H_2_O_2_ [14]. Therefore, pMSCs are potential candidates for cellular therapy to repair endothelial dysfunction and prevent complications associated with diabetes, such as thrombosis and atherosclerosis. Here, we examined the ability of pMSCs to protect the functions of endothelial cells from injury induced by glucose; an oxidative stress mediator.

First, we showed that pMSCs retain their survival and proliferation potentials in a glucose environment, which contains up to 200 mM glucose (Fig. 3a–c). pMSCs are usually found in close and continuous contact with fetal circulation, and therefore are exposed to relatively low levels of oxidative stress mediators [17, 18]. This may explain the inability of pMSCs to overcome the toxicity of glucose at high concentrations since we recently reported that exposure of pMSCs to a concentration of H_2_O_2_ higher than 200 μM is toxic [14]. These data indicate that pMSCs can resist the effects of glucose and maintain their normal function, but only up to a certain concentration, after which glucose becomes toxic.

Next, we demonstrated the ability of endothelial cells to survive in a glucose environment, but at a concentration of up to 100 mM glucose. At this concentration, the viability of endothelial cells was higher than 95%, but with reduced proliferation potential (Fig. 3d–f). This inhibitory effect of glucose on endothelial cell proliferation [19, 20] was reversed by pMSCs (Fig. 4a–c). Interestingly, pMSCs showed a dual effect on the reversibility of glucose-treated endothelial cells (Fig. 4f). Molecules produced by unstimulated pMSCs (CMpMSC) have a reversible effect on glucose-treated endothelial cells while the intercellular direct contact (ICpMSC) with endothelial cells showed an irreversible stimulatory effect on glucose-treated endothelial cells (Fig. 4f). These data provide evidence that pMSCs protect endothelial cell proliferation from the negative effect of glucose. This is supported by the finding that pMSCs modify the expression of genes mediating endothelial cell proliferation. CMpMSC and ICpMSC reduced endothelial cell expression of antiproliferative genes including *CAV1 *[21] and *COL18A1* [22] (Tables 2 and 5). In addition, ICpMSC induced endothelial cell expression of pro-proliferative genes including *PLAT* [20] and *PDGFRA* [23] (Table 5). This protective role of pMSCs was further confirmed by the ability of CMpMSC to reverse the inhibitory effect of glucose on endothelial cell migration [24] (Fig. 6a), and this stimulatory effect of pMSCs (CMpMSC and ICpMSC) on endothelial cell migration is irreversible (Fig. 6c). One possibility is that pMSCs induce glucose-treated endothelial cell migration by upregulating their expression of pro-migratory genes including *EDN1* [25], *PF4* [26], *ICAM1* [23], and *SPHK1* [27] (Tables 3 and 6).

We previously reported that pMSCs produce many molecules with survival, pro-proliferative, and migratory activities, such as *IL-6* [28], *IL-8 *[29, 30], *IL-10* [31], *IL-11* [32], and *PDGF-Rβ* [33]. These molecules potentially mediate the pMSC protective effects on endothelial cells treated with glucose. However, future functional studies are essential to elucidate the detailed molecular mechanism.

Migration is an important early step in endothelial cell biology that is followed by angiogenesis [15]. In this study, CMpMSC do not interfere with the angiogenic activity of endothelial cells (Fig. 8b). Importantly, CMpMSC reversed the anti-angiogenic effect of glucose [20] on endothelial cells (Fig. 8d, e). This protective function of CMpMSC on endothelial cell angiogenesis could be mediated by a number of angiogenic genes. CMpMSC reduced glucose-treated endothelial cell expression of various anti-angiogenic genes including *AGT* [34] and *COL18A1* [22] (Table 3). In addition, CMpMSC increased glucose-treated endothelial cell expression of various pro-angiogenic genes including *F2R* [35], *ICAM1* [23], *PGF* [36], and *CCL2* [37] (Table 3). In contrast, ICpMSC inhibited endothelial cell angiogenesis and did not prevent the anti-angiogenic effect of glucose on endothelial cells (Fig. 8f). This anti-angiogenic effect of pMSCs is possibly mediated by a number of anti-angiogenic genes including *TIMP-1* [38], *PF4* [26], and *CASP1* [39] (Table 6), although ICpMSC also reduced glucose-treated endothelial cell expression of anti-angiogenic genes including *AGT* [34] and *COL18A1 *[22] (Table 6) and induced the expression of pro-angiogenic genes including *F2R* [35], *ICAM1* [23], *PGF* [36], *CCL2* [37], and *PTGS2* [40] by endothelial cells (Table 6). These data provide evidence that pMSCs have dual effects (i.e. “a double-edged sword”) on the angiogenic activity of endothelial cells as was previously reported for the immunomodulatory properties of bone marrow-derived MSCs [41]. Supporting evidence comes from the ability of pMSCs to produce both anti-angiogenic molecules, such as *IL-12* [42], and pro-angiogenic molecules such as *IL-8* [29] and *IL-10* [43]. Our data indicate that the nature of pMSC treatments determines the type of angiogenic activity of pMSCs on endothelial cells. However, future study is essential to determine what factors determine whether the effects are pro-angiogenic or anti-angiogenic.

The exposure of endothelial cells to high levels of glucose increases their permeability [44]. This in turn enhances the infiltration of monocytes through endothelium as reported previously [44]. pMSCs reduced the stimulatory effect of glucose on monocyte infiltration through the endothelial cell monolayer, which suggests that pMSCs reduce the stimulatory effect of glucose on endothelial cell permeability (Fig. 7a). The preconditioning of endothelial cells with glucose also increased endothelial cell permeability, an effect that was reversed by ICpMSC but not by CMpMSC (Fig. 7b). This further shows the dual effects of pMSCs on the reversibility of endothelial cells in the presence of glucose. These data further support a protective effect of pMSCs on endothelial cells. The addition of pMSCs (CMpMSC or ICpMSC) to glucose-treated endothelial cells downregulated and upregulated endothelial cell expression of pro-permeability genes (i.e. *ALOX5* [45] and *NPPB* [46]) and the anti-permeability genes (i.e. *NPR1* [47]), respectively (Tables 4 and 7). In addition, pMSCs reduced the glucose stimulatory effect on endothelial cell expression of genes (ENG [48], *VCAM1* [49]) which mediate the infiltration of monocytes into endothelium (Tables 4 and 7). As for angiogenesis, pMSCs (CMpMSC and ICpMSC) showed a dual effect on endothelial expression of pro-invasion (*ICAM1*, *IL1β*, and *IL6*), anti-invasion (*CAV1*), and pro-infiltration (*SELL* and *SELE*) genes (Tables 4 and 7). Together, these data provide evidence of multiple protective roles that pMSCs have on the permeability of endothelial cells via mechanisms that involve the genes indicated. This protective role of pMSCs was further supported by the ability of pMSCs to induce glucose-treated endothelial cell expression of various genes mediating their survival (i.e. *EDN1* [50, 51], *TYMP *[52], *PROCR* [53], *F2R* [35, 53], *TGFB1* [54], *BCL2L1* [55], *MMP1* [56], *KDR* [57], *SPHK1* [58], *TNFSF10* [59]) (Tables 2 and 5). Finally, pMSCs reduced glucose-treated endothelial cell expression of genes that induce their apoptosis (*FASLG* [60]), injury (*ENG* [48], *CX3CL1* [61], *F3* [62], *THBD* [62], *AGTR1* [63]), and inflammation (*IL3* [64], *ALOX5* [65], *FLT1* [66]) (Tables 2 and 5). These data further support pMSCs having a beneficial effect on multiple endothelial cell functions in the presence of glucose.

## Conclusions

**Fig. 9:**
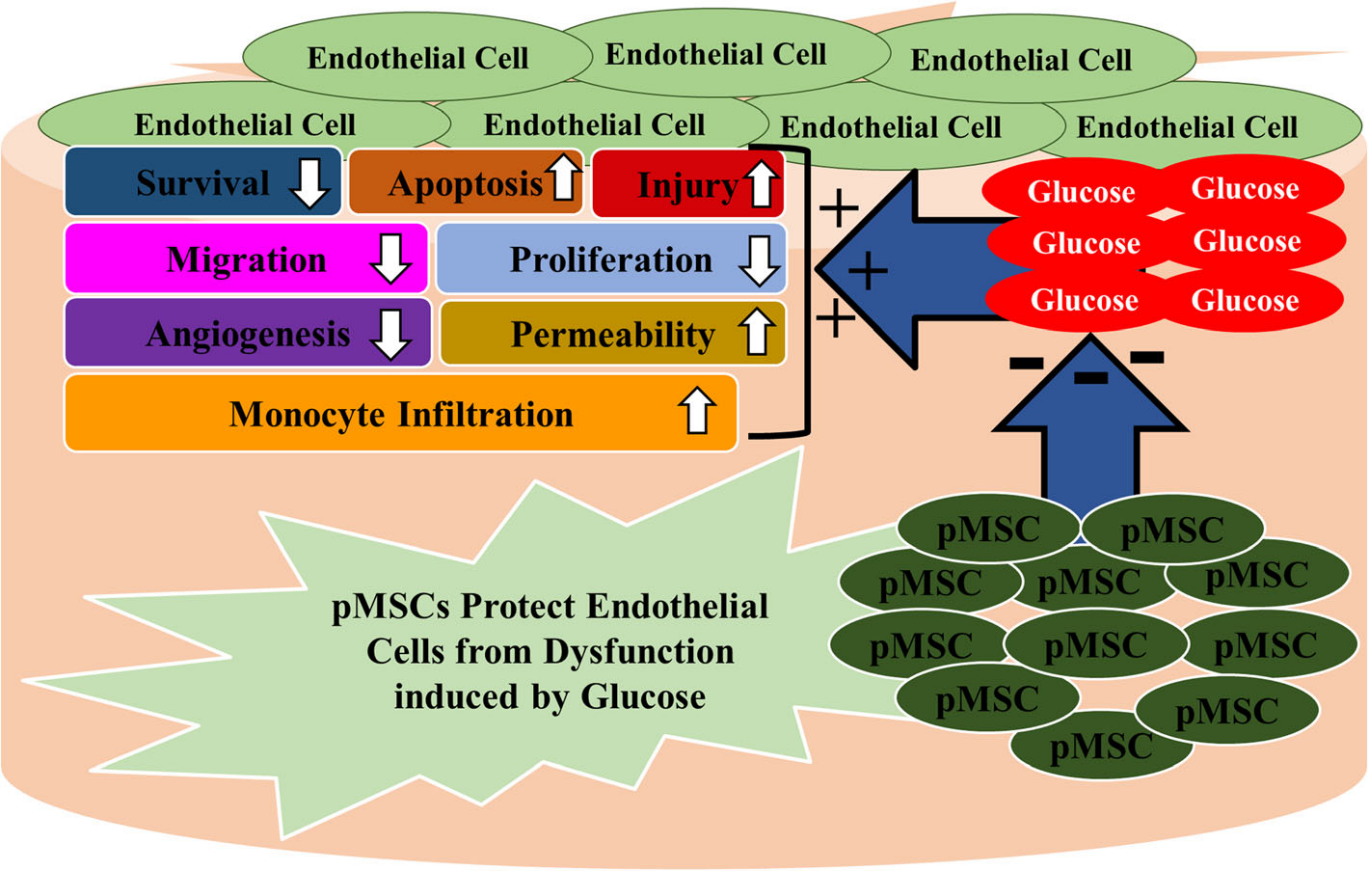
Proposed effects of placental mesenchymal stem cells (pMSCs) on modifying negative impact of glucose on endothelial cell functions. pMSCs prevent endothelial cell injury and apoptosis from glucose and reverse inhibitory effects of glucose on endothelial cell survival, proliferation, migration, and angiogenesis. pMSCs also reduce stimulatory effects of glucose on endothelial cell permeability and monocyte infiltration through endothelial cells

This is the first comprehensive study to provide evidence for a protective role of pMSCs on endothelial cells in an oxidative stress environment induced by glucose. pMSCs protect important functions of endothelial cells (i.e. proliferation, migration, angiogenesis, and permeability) from the negative impact of glucose (Fig. 9). Endothelial cell injury is a hallmark of vascular diseases, such as diabetes, that results in adverse complications leading to thrombosis and atherosclerosis. Therefore, we propose that pMSCs are promising candidates for stem cell-based therapies to treat vascular injury and the adverse complications associated with inflammatory diseases, such as diabetes and cardiovascular diseases. However, the therapeutic value of pMSCs needs to be determined in future animal studies.

## References

[1] Prentki M, Nolan CJ (2006). Islet beta cell failure in type 2 diabetes. J Clin Invest.

[2] Beckman JA, Creager MA, Libby P (2002). Diabetes and atherosclerosis: epidemiology, pathophysiology, and management. JAMA.

[3] Morel O, Kessler L, Ohlmann P, Bareiss P (2010). Diabetes and the platelet: toward new therapeutic paradigms for diabetic atherothrombosis. Atherosclerosis.

[4] Ferreiro JL, Angiolillo DJ (2011). Diabetes and antiplatelet therapy in acute coronary syndrome. Circulation.

[5] Paneni F, Beckman JA, Creager MA, Cosentino F (2013). Diabetes and vascular disease: pathophysiology, clinical consequences, and medical therapy: part I. Eur Heart J.

[6] Giacco F, Brownlee M (2010). Oxidative stress and diabetic complications. Circ Res.

[7] Tabit CE, Chung WB, Hamburg NM, Vita JA (2010). Endothelial dysfunction in diabetes mellitus: molecular mechanisms and clinical implications. Rev Endocr Metab Disord.

[8] Carr ME (2001). Diabetes mellitus: a hypercoagulable state. J Diabetes Complicat.

[9] Emerging Risk Factors C, Di Angelantonio E, Kaptoge S, Wormser D, Willeit P, Butterworth AS (2015). Association of cardiometabolic multimorbidity with mortality. JAMA.

[10] Booth GL, Kapral MK, Fung K, Tu JV (2006). Relation between age and cardiovascular disease in men and women with diabetes compared with non-diabetic people: a population-based retrospective cohort study. Lancet.

[11] Abumaree MH, Al Jumah MA, Kalionis B, Jawdat D, Al Khaldi A, AlTalabani AA (2013). Phenotypic and functional characterization of mesenchymal stem cells from chorionic villi of human term placenta. Stem Cell Rev.

[12] Abumaree MH, Al Jumah MA, Kalionis B, Jawdat D, Al Khaldi A, Abomaray FM (2013). Human placental mesenchymal stem cells (pMSCs) play a role as immune suppressive cells by shifting macrophage differentiation from inflammatory M1 to anti-inflammatory M2 macrophages. Stem Cell Rev.

[13] Abomaray FM, Al Jumah MA, Kalionis B, AlAskar AS, Al Harthy S, Jawdat D (2015). Human chorionic villous mesenchymal stem cells modify the functions of human dendritic cells, and induce an anti-inflammatory phenotype in CD1+ dendritic cells. Stem Cell Rev.

[14] Abumaree MH, Hakami M, Abomaray FM, Alshabibi MA, Kalionis B, Al Jumah MA (2017). Human chorionic villous mesenchymal stem/stromal cells modify the effects of oxidative stress on endothelial cell functions. Placenta.

[15] Alshabibi MA, Al Huqail AJ, Khatlani T, Abomaray FM, Alaskar AS, Alawad AO (2017). Mesenchymal stem/multipotent stromal cells from human decidua basalis reduce endothelial cell activation. Stem Cells Dev.

[16] Abomaray FM, Al Jumah MA, Alsaad KO, Jawdat D, Al Khaldi A, AlAskar AS (2016). Phenotypic and functional characterization of mesenchymal stem/multipotent stromal cells from decidua basalis of human term placenta. Stem Cells Int.

[17] Braekke K, Harsem NK, Staff AC (2006). Oxidative stress and antioxidant status in fetal circulation in preeclampsia. Pediatr Res.

[18] Kusuma GD, Abumaree MH, Pertile MD, Perkins AV, Brennecke SP, Kalionis B (2016). Mesenchymal stem/stromal cells derived from a reproductive tissue niche under oxidative stress have high aldehyde dehydrogenase activity. Stem Cell Rev.

[19] Stout RW (1982). Glucose inhibits replication of cultured human endothelial cells. Diabetologia.

[20] Moriya J, Ferrara N (2015). Inhibition of protein kinase C enhances angiogenesis induced by platelet-derived growth factor C in hyperglycemic endothelial cells. Cardiovasc Diabetol.

[21] Fang K, Fu W, Beardsley AR, Sun X, Lisanti MP, Liu J (2007). Overexpression of caveolin-1 inhibits endothelial cell proliferation by arresting the cell cycle at G0/G1 phase. Cell Cycle.

[22] Clement B, Musso O, Lietard J, Theret N (1999). Homeostatic control of angiogenesis: a newly identified function of the liver?. Hepatology.

[23] Radisavljevic Z, Avraham H, Avraham S (2000). Vascular endothelial growth factor up-regulates ICAM-1 expression via the phosphatidylinositol 3 OH-kinase/AKT/nitric oxide pathway and modulates migration of brain microvascular endothelial cells. J Biol Chem.

[24] Hamuro M, Polan J, Natarajan M, Mohan S (2002). High glucose induced nuclear factor kappa B mediated inhibition of endothelial cell migration. Atherosclerosis.

[25] Daher Z, Noel J, Claing A (2008). Endothelin-1 promotes migration of endothelial cells through the activation of ARF6 and the regulation of FAK activity. Cell Signal.

[26] Sarabi A, Kramp BK, Drechsler M, Hackeng TM, Soehnlein O, Weber C (2011). CXCL4L1 inhibits angiogenesis and induces undirected endothelial cell migration without affecting endothelial cell proliferation and monocyte recruitment. J Thromb Haemost.

[27] Paik JH, Chae S, Lee MJ, Thangada S, Hla T (2001). Sphingosine 1-phosphate-induced endothelial cell migration requires the expression of EDG-1 and EDG-3 receptors and rho-dependent activation of alpha vbeta3- and beta1-containing integrins. J Biol Chem.

[28] Yao JS, Zhai W, Young WL, Yang GY (2006). Interleukin-6 triggers human cerebral endothelial cells proliferation and migration: the role for KDR and MMP-9. Biochem Biophys Res Commun.

[29] Li A, Dubey S, Varney ML, Dave BJ, Singh RK (2003). IL-8 directly enhanced endothelial cell survival, proliferation, and matrix metalloproteinases production and regulated angiogenesis. J Immunol.

[30] Lai Y, Shen Y, Liu XH, Zhang Y, Zeng Y, Liu YF (2011). Interleukin-8 induces the endothelial cell migration through the activation of phosphoinositide 3-kinase-Rac1/RhoA pathway. Int J Biol Sci.

[31] Verma SK, Garikipati VN, Krishnamurthy P, Khan M, Thorne T, Qin G (2016). IL-10 accelerates re-endothelialization and inhibits post-injury intimal hyperplasia following carotid artery denudation. PLoS One.

[32] Kirkiles-Smith NC, Mahboubi K, Plescia J, McNiff JM, Karras J, Schechner JS (2004). IL-11 protects human microvascular endothelium from alloinjury in vivo by induction of survivin expression. J Immunol.

[33] Heldin CH, Ostman A, Ronnstrand L (1998). Signal transduction via platelet-derived growth factor receptors. Biochim Biophys Acta.

[34] Celerier J, Cruz A, Lamande N, Gasc JM, Corvol P (2002). Angiotensinogen and its cleaved derivatives inhibit angiogenesis. Hypertension.

[35] Zania P, Kritikou S, Flordellis CS, Maragoudakis ME, Tsopanoglou NE (2006). Blockade of angiogenesis by small molecule antagonists to protease-activated receptor-1: association with endothelial cell growth suppression and induction of apoptosis. J Pharmacol Exp Ther.

[36] Ribatti D (2008). The discovery of the placental growth factor and its role in angiogenesis: a historical review. Angiogenesis.

[37] Niu J, Wang K, Zhelyabovska O, Saad Y, Kolattukudy PE (2013). MCP-1-induced protein promotes endothelial-like and angiogenic properties in human bone marrow monocytic cells. J Pharmacol Exp Ther.

[38] Reed MJ, Koike T, Sadoun E, Sage EH, Puolakkainen P (2003). Inhibition of TIMP1 enhances angiogenesis in vivo and cell migration in vitro. Microvasc Res.

[39] Lopez-Pastrana J, Ferrer LM, Li YF, Xiong X, Xi H, Cueto R (2015). Inhibition of Caspase-1 activation in endothelial cells improves angiogenesis: a novel therapeutic potential for ischemia. J Biol Chem.

[40] He T, Lu T, d'Uscio LV, Lam CF, Lee HC, Katusic ZS (2008). Angiogenic function of prostacyclin biosynthesis in human endothelial progenitor cells. Circ Res.

[41] Li W, Ren G, Huang Y, Su J, Han Y, Li J (2012). Mesenchymal stem cells: a double-edged sword in regulating immune responses. Cell Death Differ.

[42] Sgadari C, Angiolillo AL, Tosato G (1996). Inhibition of angiogenesis by interleukin-12 is mediated by the interferon-inducible protein 10. Blood.

[43] Dace DS, Khan AA, Kelly J, Apte RS (2008). Interleukin-10 promotes pathological angiogenesis by regulating macrophage response to hypoxia during development. PLoS One.

[44] Zhao XY, Wang XF, Li L, Zhang L, Shen DL, Li DH (2015). Effects of high glucose on human umbilical vein endothelial cell permeability and myosin light chain phosphorylation. Diabetol Metab Syndr.

[45] Valdivielso JM, Montero A, Badr KF, Munger KA (2003). Inhibition of 5-lipoxygenase activating protein decreases proteinuria in diabetic rats. J Nephrol.

[46] Chen W, Gassner B, Borner S, Nikolaev VO, Schlegel N, Waschke J (2012). Atrial natriuretic peptide enhances microvascular albumin permeability by the caveolae-mediated transcellular pathway. Cardiovasc Res.

[47] Furst R, Bubik MF, Bihari P, Mayer BA, Khandoga AG, Hoffmann F (2008). Atrial natriuretic peptide protects against histamine-induced endothelial barrier dysfunction in vivo. Mol Pharmacol.

[48] Rossi E, Sanz-Rodriguez F, Eleno N, Duwell A, Blanco FJ, Langa C (2013). Endothelial endoglin is involved in inflammation: role in leukocyte adhesion and transmigration. Blood.

[49] Esposito C, Fasoli G, Plati AR, Bellotti N, Conte MM, Cornacchia F (2001). Long-term exposure to high glucose up-regulates VCAM-induced endothelial cell adhesiveness to PBMC. Kidney Int.

[50] Dong F, Zhang X, Wold LE, Ren Q, Zhang Z, Ren J (2005). Endothelin-1 enhances oxidative stress, cell proliferation and reduces apoptosis in human umbilical vein endothelial cells: role of ETB receptor, NADPH oxidase and caveolin-1. Br. Aust J Pharm.

[51] Mikhail M, Vachon PH, D'Orleans-Juste P, Jacques D, Bkaily G (2017). Role of endothelin-1 and its receptors, ETA and ETB, in the survival of human vascular endothelial cells. Can J Physiol Pharmacol.

[52] Sengupta S, Sellers LA, Matheson HB, Fan TP (2003). Thymidine phosphorylase induces angiogenesis in vivo and in vitro: an evaluation of possible mechanisms. Br J Pharmacol.

[53] Bock F, Shahzad K, Wang H, Stoyanov S, Wolter J, Dong W (2013). Activated protein C ameliorates diabetic nephropathy by epigenetically inhibiting the redox enzyme p66Shc. Proc Natl Acad Sci U S A.

[54] Vinals F, Pouyssegur J (2001). Transforming growth factor beta1 (TGF-beta1) promotes endothelial cell survival during in vitro angiogenesis via an autocrine mechanism implicating TGF-alpha signaling. Mol Cell Biol.

[55] Kern TS, Du Y, Miller CM, Hatala DA, Levin LA (2010). Overexpression of Bcl-2 in vascular endothelium inhibits the microvascular lesions of diabetic retinopathy. Am J Pathol.

[56] Mazor R, Alsaigh T, Shaked H, Altshuler AE, Pocock ES, Kistler EB (2013). Matrix metalloproteinase-1-mediated up-regulation of vascular endothelial growth factor-2 in endothelial cells. J Biol Chem.

[57] Wang JF, Zhang X, Groopman JE (2004). Activation of vascular endothelial growth factor receptor-3 and its downstream signaling promote cell survival under oxidative stress. J Biol Chem.

[58] Kwon YG, Min JK, Kim KM, Lee DJ, Billiar TR, Kim YM (2001). Sphingosine 1-phosphate protects human umbilical vein endothelial cells from serum-deprived apoptosis by nitric oxide production. J Biol Chem.

[59] Secchiero P, Gonelli A, Carnevale E, Milani D, Pandolfi A, Zella D (2003). TRAIL promotes the survival and proliferation of primary human vascular endothelial cells by activating the Akt and ERK pathways. Circulation.

[60] Joussen AM, Poulaki V, Mitsiades N, Cai WY, Suzuma I, Pak J (2003). Suppression of Fas-FasL-induced endothelial cell apoptosis prevents diabetic blood-retinal barrier breakdown in a model of streptozotocin-induced diabetes. FASEB J.

[61] Li G, Xu Y, Sheng X, Liu H, Guo J, Wang J (2017). Naringin protects against high glucose-induced human endothelial cell injury via antioxidation and CX3CL1 downregulation. Cell Physiol Biochem.

[62] Stehouwer CD (2004). Endothelial dysfunction in diabetic nephropathy: state of the art and potential significance for non-diabetic renal disease. Nephrol Dial Transplant.

[63] Willemsen JM, Westerink JW, Dallinga-Thie GM, van Zonneveld AJ, Gaillard CA, Rabelink TJ (2007). Angiotensin II type 1 receptor blockade improves hyperglycemia-induced endothelial dysfunction and reduces proinflammatory cytokine release from leukocytes. J Cardiovasc Pharmacol.

[64] Brizzi MF, Garbarino G, Rossi PR, Pagliardi GL, Arduino C, Avanzi GC (1993). Interleukin 3 stimulates proliferation and triggers endothelial-leukocyte adhesion molecule 1 gene activation of human endothelial cells. J Clin Invest.

[65] Sasson S, Davarashvili A, Reich R (1999). Role of lipoxygenase in the regulation of glucose transport in aortic vascular cells. Adv Exp Med Biol.

[66] Yang KS, Lim JH, Kim TW, Kim MY, Kim Y, Chung S (2014). Vascular endothelial growth factor-receptor 1 inhibition aggravates diabetic nephropathy through eNOS signaling pathway in db/db mice. PLoS One.

